# Graded gene expression changes determine phenotype severity in mouse models of *CRX*-associated retinopathies

**DOI:** 10.1186/s13059-015-0732-z

**Published:** 2015-09-01

**Authors:** Philip A. Ruzycki, Nicholas M. Tran, Vladimir J. Kefalov, Alexander V. Kolesnikov, Shiming Chen

**Affiliations:** Department of Ophthalmology and Visual Sciences, Washington University School of Medicine, Saint Louis, MO USA; Department of Developmental Biology, Washington University School of Medicine, Saint Louis, MO USA; Molecular Genetics and Genomics Graduate Program, Division of Biology & Biomedical Sciences, Washington University School of Medicine, Saint Louis, MO USA; Current address: Department of Molecular and Cellular Biology, Harvard University, NW 335.30, 52 Oxford St, Cambridge, MA 02138 USA

## Abstract

**Background:**

Mutations in the cone-rod-homeobox protein CRX are typically associated with dominant blinding retinopathies with variable age of onset and severity. Five well-characterized mouse models carrying different *Crx* mutations show a wide range of disease phenotypes. To determine if the phenotype variability correlates with distinct changes in CRX target gene expression, we perform RNA-seq analyses on three of these models and compare the results with published data.

**Results:**

Despite dramatic phenotypic differences between the three models tested, graded expression changes in shared sets of genes are detected. Phenotype severity correlates with the down-regulation of genes encoding key rod and cone phototransduction proteins. Interestingly, in increasingly severe mouse models, the transcription of many rod-enriched genes decreases decrementally, whereas that of cone-enriched genes increases incrementally. Unlike down-regulated genes, which show a high degree of CRX binding and dynamic epigenetic profiles in normal retinas, the up-regulated cone-enriched genes do not correlate with direct activity of CRX, but instead likely reflect a change in rod cell-fate integrity. Furthermore, these analyses describe the impact of minor gene expression changes on the phenotype, as two mutants showed marginally distinguishable expression patterns but huge phenotypic differences, including distinct mechanisms of retinal degeneration.

**Conclusions:**

Our results implicate a threshold effect of gene expression level on photoreceptor function and survival, highlight the importance of CRX in photoreceptor subtype development and maintenance, and provide a molecular basis for phenotype variability in *CRX*-associated retinopathies.

**Electronic supplementary material:**

The online version of this article (doi:10.1186/s13059-015-0732-z) contains supplementary material, which is available to authorized users.

## Background

Rod and cone photoreceptors are the two primary light detecting cell types of the retina and are essential for vision. Each cell type preferentially expresses a set of genes critical for development and maintenance of its specialized function. This cell type-specific gene expression is regulated by a network of transcription factors (reviewed in [1, 2]). Disruptions in this regulatory network can have a dramatic effect on rod [3–6] and cone [7, 8] development, function, cell-fate integrity and survival. The homeodomain transcription factor CRX (Cone-rod homeobox protein) is expressed in both rods and cones and plays a central role in mediating photoreceptor transcription. CRX works with rod-specific, cone-specific and general transcription factors to control photoreceptor gene expression [9–14]. In particular, CRX and the rod-specific transcription factor NRL (Neural retina leucine zipper protein) cooperatively regulate rod gene transcription and have highly overlapping DNA-binding patterns [15–17]. Loss of CRX dramatically impairs both rod and cone photoreceptor gene transcription, leading to failed photoreceptor maturation and rapid degeneration [18], while the loss of NRL converts rods into cells with cone-like transcription and functional properties [3, 16].

Mutations in the human *CRX* gene have been associated with dominant forms of retinal degenerative diseases such as retinitis pigmentosa, cone-rod dystrophy (CoRD) and Leber congenital amaurosis (LCA), with varied age of onset and severity (reviewed in [14, 19, 20]). Disease-causing mutations fall into at least four classes, based on mutation type, pathogenic mechanism and molecular properties of the mutant protein: I) hypomorphic substitution mutations with reduced DNA-binding activity; II) antimorphic substitution mutations with variable DNA-binding; III) antimorphic frameshift mutations with intact DNA-binding; and IV) antimorphic frameshift mutations with variable DNA-binding (reviewed in [14]). While each class of disease-causing mutation is associated with distinct clinical phenotypes, the underlying transcriptional changes mediating these phenotypes are poorly understood.

**Fig. 1:**
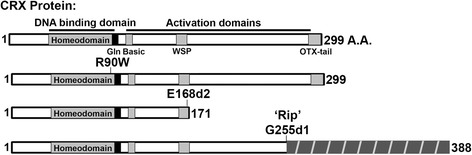
Schematic diagram of wild-type (WT) and mutant CRX proteins made by the indicated mouse models. The WT CRX protein shown on the top is 299 amino acids (*A.A.*) in length and contains the indicated DNA-binding and transactivation domains (indicated by *bars* above diagram) and several conserved motifs (marked by *solid grey* and *black boxes*). The substitution mutation *R90W* lies within the homeodomain and reduces DNA-binding [21]. The frameshift mutation *E168d2* results in a C-terminus truncated CRX protein that retains DNA-binding capability but fails to activate transcription, and is, therefore, antimorphic [21]. The frameshift mutation *G255d1* ‘*Rip*’ results in a non-homologous C-terminal extension (*dark grey hashed box*), creating an antimorphic protein that no longer binds DNA [24]. The phenotypes of heterozygous mice carrying each of these mutations are summarized in Table 1

In this study, we used mRNA sequencing (RNA-seq) to examine gene expression in the developing and adult retinas of three *Crx* mutation knock-in mouse models that have distinct retinal phenotypes [21]: *Crx*^*R90W*^ (*R90W*), *Crx*^*E168d2*^ (*E168d2*) and *Crx*^*E168d2neo*^ (*E168d2neo*) (Fig. 1, Table 1)*.* The *R90W* mice carry a class I mutation and their phenotypes resemble mild late-onset dominant CoRD and recessive LCA. *E168d2* and *E168d2neo* mice carry the same class III mutation but *E168d2neo* mice express the mutant protein at a lower level, due to the retention of an intronic *neomycin* cassette. *E168d2* mouse phenotypes resemble severe dominant LCA, while *E168d2neo* mouse phenotypes resemble a less severe dominant CoRD phenotype, due to reduced mutant protein expression. We assessed how each *Crx* mutation impacted the expression of retinal cell type-specific genes and investigated cellular pathways that may contribute to disease. We correlated these transcriptional changes with the direct DNA-binding activity of CRX [15] and NRL [17] and the epigenetic landscape of rods and cones [22, 23]. Additionally, we compared the expression profiles of these *Crx* knock-in mice to the previously characterized *Crx−/−* and *Crx*^*Rip*^ (*Rip*) mouse models. *Rip* mice carry a class IV mutation and have a more severe dominant LCA phenotype than *E168d2* mice [24]. Our results demonstrate that all five mouse models have graded changes in photoreceptor-specific gene expression that correlate with the severity of their phenotypes. These graded changes include reduced expression of direct CRX target genes required for rod/cone photoreceptor function and survival, and derepression of ‘cone’ genes in rods through an indirect mechanism, suggesting that rod cell-fate integrity is compromised in the more severe models.

**Table 1:** Phenotype summary of heterozygous *Crx* mutant mice

Mouse^a^	Mutation class^b^	CRX expression^c^	Rod		Cone		Disease model	Phenotype severity^e^
			Function^d^	Degeneration	Function^d^	Degeneration		
WT	NA	+	++++	Undetectable	++++	Undetectable	NA	NA
*R90W*	I	+	++++	Undetectable	++++	Undetectable	CoRD	Mild
*E168d2neo*	III	+	+++	Undetectable	++	≥1 year	CoRD	Moderate
*E168d2*	III	++	++	1–6 months	+	1 month	LCA	Severe
*Rip*	IV	+	-	1–18 months	-	Undetectable	LCA	Very severe

^a^ Heterozygous mice harboring the indicated *Crx* mutations [21, 24] are used for phenotype comparisons

^b^ Classification described by [14]

^c^ Grading based on quantitative western blots [21]. Note a twofold increase in *E168d2* but normal level in others

^d^ Grading based on reduction of electroretinography (ERG) peak amplitudes [21]. Dashes indicate undetected ERG signals

^e^ Severity based on combined morphological and functional deficits

WT *C57BL/6 J* wild-type control, *NA* not applicable

Lastly, we identified threshold effects of gene expression changes on retinal phenotype. Despite having only small differences in gene expression changes, heterozygous *E168d2* and *E168d2neo* mice showed drastically different phenotypes, including light-independent versus light-dependent photoreceptor degeneration, respectively. These phenotypic and mechanistic differences between the two models are likely attributed to slight shifts in gene expression throughout several photoreceptor-specific pathways, particularly phototransduction and the retinoid (visual) cycle. This highlights the delicate balance between photoreceptor gene transcription, function and cellular integrity. These results demonstrate that the transcriptional landscape in models of retinal degeneration can dramatically affect disease pathology. Effective therapeutic design may therefore be highly context specific.

## Results

### *Crx* mutations cause graded expression changes in shared gene sets, correlating with phenotype severity

To assess the effects of *Crx* mutations on retinal gene expression we performed RNA-seq on retinas of the *Crx* mutant mouse models *R90W*, *E168d2* and *E168d2neo*, and age-matched wild-type (WT) controls, in triplicate. As listed in Table 2, we analyzed heterozygous mutants at both postnatal day (P)10 and P21, but homozygous mutants at P10 only, as their retinas are severely degenerated at later ages. Because they lack WT CRX to antagonize the antimorphic mutant CRX protein, homozygous *E168d2* and *E168d2neo* mice show essentially the same severe phenotype at morphological, functional and gene expression levels [21] (Table S1a in Additional file 1). Thus, we only performed RNA-seq on *E168d2/d2* to provide a reference for an extremely severe phenotype. RNA-seq libraries were sequenced on the Illumina HiSeq2000 and each generated more than 28 million mapped reads (Additional file 2). Sample quality was assessed by principal component analysis (PCA) of the expression values of all genes that passed the 5 counts per million (CPM) threshold in any genotype (Additional file 3), and by visual inspection of mapped reads at individual gene loci using Integrated Genomics Viewer (IGV) [25] (Figure S2a, b in Additional file 4). PCA plots (Additional file 3) show that the genotypes clustered as expected with the *E168d2/d2* samples showing the most variation from WT controls at both ages tested. At P10 *E168d2/+*, *E168d2neo/+* and *R90W/W* samples clustered between WT and *E168d2/d2*, corresponding to their intermediate phenotypes. In contrast, *R90W/+* samples clustered with WT samples, consistent with their normal phenotype at this age. At P21, despite increased replicate variability, differences between heterozygous mutant genotypes and WT are consistent with their phenotypes. Since the PCA analysis only described sample differences as a whole, we further determined the relationship of gene expression changes and phenotype differences, using multiple independent analyses of gene expression. First, to determine differential expression, EdgeR [26] was used to compare the triplicate samples. Changes considered “significant” were twofold or greater [fold change (FC) ≥ 2] with a false discovery rate (FDR) ≤ 0.05 from the appropriate WT control, unless otherwise noted. Additional filtering and analysis details can be found in the "Materials and methods" section. Expression changes in subsets of RNA-seq-identified genes were further validated using quantitative real time PCR (qRT-PCR; Table S1a–c in Additional file 1).

**Table 2:** List of RNA-seq experiments and number of genes with altered expression

Mouse^a^	Experiments and replicates^b^		Down-regulated genes^c^		Up-regulated genes^c^	
	P10	P21	P10	P21	P10	P21
WT	3	3	NA	NA	NA	NA
*E168d2/d2*	3	Not tested	425	NA	248	NA
*R90W/W*	3	Not tested	195	NA	70	NA
*E168d2/+*	3	3	136	150	61	43
*E168d2neo/+*	3	3	85	83	38	19
*R90W/+*	3	3	20	27	12	27

^a^ All mutant mice were backcrossed to *C57BL/6 J* (WT) control for >10 generations and genotyped for common variants [21]

^b^ Numbers represent biological replicates. Each replicate contains four pooled retinas from a male and female pair

^c^ Numbers represent transcripts significantly altered (FC ≥ 2, FDR ≤ 0.05) relative to WT control

*NA* not applicable

#### Homozygous *Crx* mutant mice show drastically changed expression of a large number of genes at P10 before photoreceptor degeneration

**Fig. 2:**
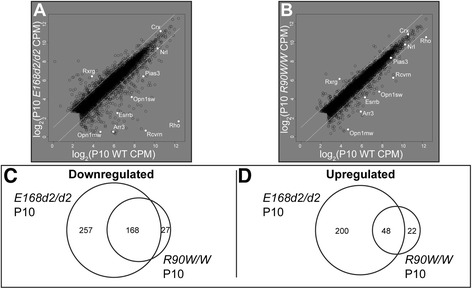
RNA-seq analyses describe overlapping sets of affected genes in P10 homozygous *Crx* mutant retinas. **a**, **b** Gene expression (log_2_ CPM) in the indicated homozygous mutants (y-axes) is compared with the WT control (*C57BL/6 J*, x-axes). Prototypical photoreceptor transcripts are labeled in white. White diagonal lines represent ±2 FC. **c**, **d** Venn diagrams illustrate the numbers of significantly affected genes that are shared or are uniquely changed in indicated mutants (FC ≥ 2 or ≤ -2, FDR ≤ 0.05)

We first compared retinal gene expression in the homozygous mutants *E168d2/d2* and *R90W/W* at P10 with that in age-matched WT controls. As shown by the scatterplots (Fig. 2a, b), both mutants displayed twofold or greater expression changes for a large number of genes. However, the number of genes affected in *E168d2/d2* was much larger than in *R90W/W* (Table 2). Among the changed genes, down-regulated genes outnumbered up-regulated genes (Table 2), consistent with the established role of CRX in the activation of transcription. Some of the affected genes (labeled in white in Fig. 2a, b) encode well-characterized proteins essential for rod and cone identity, function and survival. Next, we compared the changed gene sets between the two mutants. Even though *E168d2/d2* and *R90W/W* have mechanistically distinct mutant proteins and phenotypes [21], the significantly affected (FC ≥ 2, FDR ≤ 0.05) genes showed a high degree of overlap for both down-regulated and up-regulated gene sets (Fig. 2c, d). In addition, for these shared genes the degree of change was generally greater in *E168d2/d2* than in *R90W/W*, as seen by visually inspecting the positions of the highlighted genes in Fig. 2a versus Fig. 2b. These changes were confirmed to be consistent between replicates by comparing the raw mapped reads for several of these genes (Figure S2a in Additional file 4). Homozygous mouse retina RNA-seq results for several genes were also consistent with previous qRT-PCR data [21] (Table S1b in Additional file 1). These data suggest that photoreceptor gene expression in *R90W/W* is less disrupted than in *E168d2/d2*, consistent with the phenotype differences between the two models.

#### Heterozygous *Crx* mutant mice have moderate expression changes in fewer genes than the homozygous mutants at P10, with few independently affected genes

**Fig. 3:**
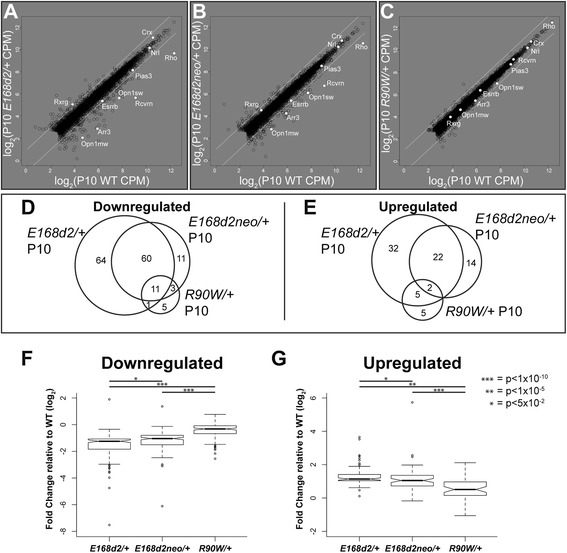
RNA-seq analyses detect graded changes in gene expression in P10 heterozygous *Crx* mutant retinas. **a**-**c** Gene expression (log_2_ CPM) in the indicated heterozygotes (y-axes) are compared with the WT control (*C57BL/6 J*, x-axes). Prototypical photoreceptor transcripts are labeled in white. White diagonal lines represent ±2 FC. **d**, **e** Venn diagrams illustrate the numbers of significantly affected genes that are shared or are uniquely changed in the indicated mutants (FC ≥ 2 or ≤ -2, FDR ≤ 0.05). **f**, **g** Analysis of the union of genes affected in heterozygous mutants (FC ≥ 2 or ≤ -2, FDR ≤ 0.05) presented as FC relative to WT control. Significance calculated by Wilcoxon rank-sum test with Bonferroni correction. Notched box whisker plot describes median and quartiles of data

To determine if heterozygous mutants also share gene expression changes between genotypes, we compared RNA-seq data from heterozygous mutants *E168d2/+*, *R90W/+*, as well as *E168d2neo/+*. At P10, the heterozygous mutants showed fewer gene changes than the respective homozygous counterparts (Table 2). Similar to the homozygous mutants, the number of significantly affected genes in heterozygotes correlated with phenotype severity in the order *E168d2/+* > *E168d2neo/+* > *R90W/+*. Figure 3a–c and Figure S2a in Additional file 4 show that, qualitatively, the white highlighted photoreceptor transcripts were most severely affected in the *E168d2/+* mutant, less affected in *E168d2neo/+* and showed no change greater than twofold in the *R90W/+* line. Furthermore, affected transcripts in heterozygotes also showed a high degree of overlap between the genotypes (Fig. 3d, e).

Next, we analyzed whether the degree of expression changes correlates with phenotype severity. To gain quantitative results for the entire datasets, we compared the overall FC (relative to WT) for the union of all affected genes (see Table 2 for total numbers) in *E168d2/+*, *E168d2neo/+* and *R90W/+* mutants (Fig. 3f, g). Both down-regulated (Fig. 3f) and up-regulated (Fig. 3g) groups showed a stepwise pattern of expression fold changes: *E168d2/+* > *E168d2neo/+* > *R90W/+*. Thus, in addition to the number of affected genes, the degree of expression changes also correlates with phenotype severity in heterozygous mutants. While this difference in expression was statistically significant (Fig. 3f, g), the differences between the median log_2_ FC for individual genes expressed in the different heterozygotes were rather small. This is in contrast to the large phenotype differences between these genotypes (Table 1), demonstrating the importance of precisely regulated gene expression for photoreceptor integrity. The expression changes determined by RNA-seq of a subset of genes were validated to be consistent between replicates (Figure S2a in Additional file 4) and accordant with previous qRT-PCR data [21] (Table S1b, c in Additional file 1).

We also directly compared the data for overlapping and independently affected genes in age-matched (P10) heterozygote and homozygote *Crx* mutant animals. Significantly fewer down-regulated and up-regulated genes were seen in the heterozygous *E168d2/+* and *R90W/+* mutants than in their homozygous counterparts (Additional file 5). Only a small fraction of genes was independently changed in either heterozygote mutant but unaffected in the homozygotes (Additional file 5). These data are consistent with the differences in phenotype severity between the heterozygous and homozygous mutants.

### *Crx* mutations specifically affect rod- and cone-enriched genes, including down-regulation of phototransduction genes

**Fig. 4:**
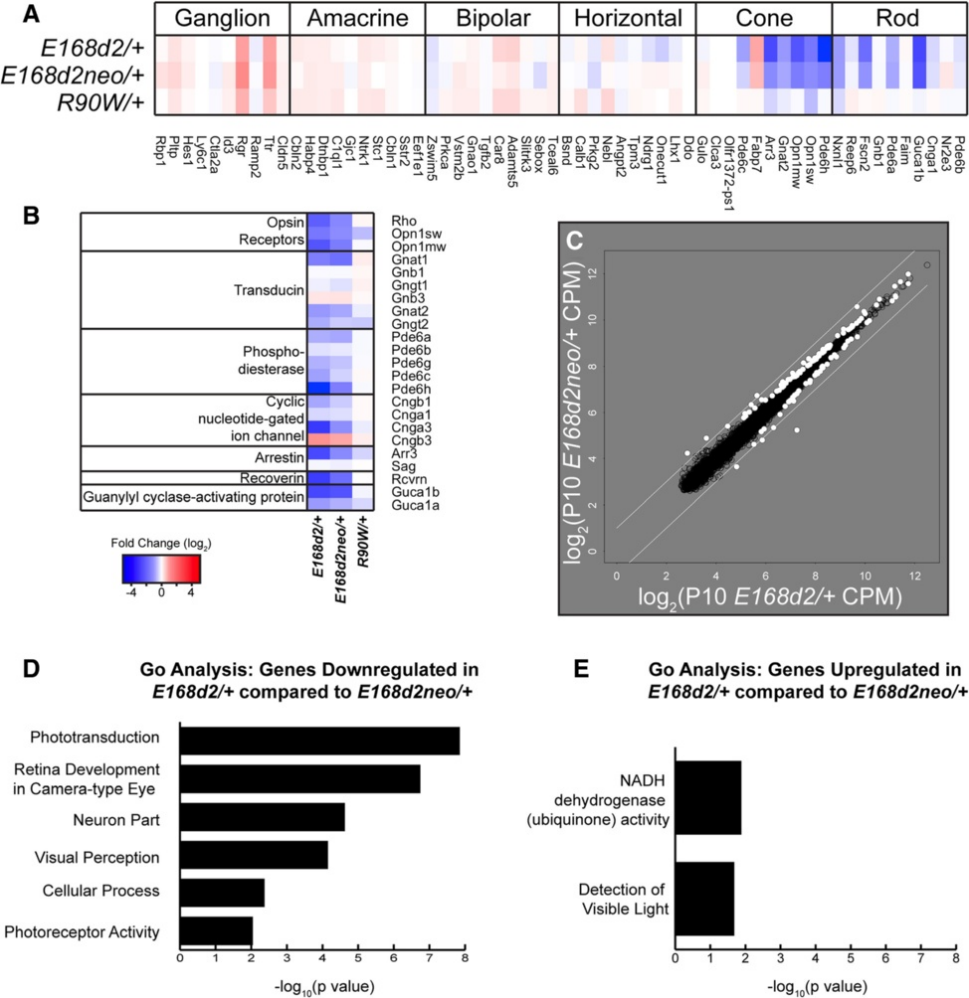
*Crx* mutant retinas show graded changes in photoreceptor-specific gene expression. **a**, **b** Heatmaps present FC of gene expression in P10 *Crx* mutants relative to that of WT age-matched controls in subsets of genes with highest cell type specificity ratios for the six retinal neurons (**a**) and photoreceptor-enriched genes involved in phototransduction (**b**). **c** Changes in gene expression levels (log_2_ CPM) are compared for *E168d2/+* (x-axis) versus *E168d2neo/+* (y-axis). Highlighted genes represent 118 transcripts significantly differently expressed between the mutants (FDR ≤ 0.05, no FC cutoff; see Additional files 7 and 11 for designations). **d**, **e** Top Gene Ontology (*GO*) terms of down-regulated (**d**) and up-regulated (**e**) gene sets in *E168d2/+* compared with *E168d2neo/+*

To ensure that decreased retinal function and impaired development of the *Crx* mutant mice are not a result of perturbations to other non-photoreceptor cell types, we analyzed the P10 RNA-seq data for changes in expression of genes representing different types of retinal neurons. For each cell type, we chose the ten genes with the highest specificity ratio [27] present in our data. The heatmap in Fig. 4a represents the FC from WT for each of these genes in the P10 mutants, organized by the cell type they represent. The cell types that showed the greatest gene expression changes in all mutants were rod and cone photoreceptors, whereas large-scale changes in gene expression were not observed in other retinal cell types at the age of P10.

We also specifically analyzed expression of genes important for retinal visual function, as changes in this pathway in cell types other than photoreceptors could account for the decreased function as measured by ERG [21]. Additional file 6 shows that non-photoreceptor-specific genes encoding proteins involved in visual function had little to no change in expression in any of the mutants. In contrast, expression changes were seen in many known photoreceptor-enriched components of the phototransduction cascade (Fig. 4b). Nearly every constituent was negatively impacted in *E168d2/+*, but remained virtually unaffected in *R90W/+. E168d2neo/+* adopted an intermediate expression level, again emphasizing the stepwise and graded changes between the three genotypes.

### Insights from *E168d2/+* and *E168d2neo/+*: small changes in gene expression level strongly influence phenotype

Since the above analysis detected little difference between the two phenotypically distinct mouse lines that both carry the same *Crx* mutation, *E168d2/+* and *E168d2neo/+*, we expanded our analyses to directly compare these two datasets. A scatterplot comparing *E168d2*/+ and *E168d2neo*/+ shows only subtle expression differences (Fig. 4c), further confirming the analyses presented in Table 2 and Fig. 3. Differences in gene expression levels were within the twofold margin with only a few exceptions. To decipher the genes that are likely to impact phenotype severity, we analyzed the 118 genes that passed the statistical threshold in the direct comparison of *E168d2/+* versus *E168d2neo/+* (FDR ≤ 0.05, no FC cutoff, represented in white in Fig. 4c). To confirm the validity of the results of this comparison, we also calculated the Z-score of each biological replicate from the mean expression level (Additional file 7), which showed reproducible differences between the two genotypes. The list of down-regulated genes in *E168d2/+* was highly enriched for those relevant to photoreceptor biology and function by Gene Ontology (GO) analyses (Fig. 4d). In contrast, the up-regulated genes showed only a modest enrichment for a single photoreceptor-relevant GO category (Fig. 4e). These findings suggest threshold effects of expression level changes on photoreceptor phenotype, especially in those pathways represented in the down-regulated gene set illustrated in Fig. 4d.

### Heterozygous mutants show normal gene expression trends from P10 to P21, but many genes fail to reach the normal level at P21

*Crx* mutant mice show early deficits in photoreceptor morphology and function [18, 21]. In heterozygous *Crx* knockout and *R90W* mice, morphology and function recover at later ages, suggesting a transient developmental delay. *E168d2neo/+* mice also recover rod morphology but have abnormal cone morphology and only partially recover rod and cone function in adulthood. *E168d2/+* mice remain impaired in adulthood with shortened outer segments and severely impaired retinal function [21], suggesting a blockade in photoreceptor maturation. To determine if differences in morphological and functional recovery are related to gene expression changes over time, we also performed RNA-seq analyses on heterozygous *R90W*, *E168d2* and *E168d2neo* mutants at P21 when photoreceptors are mature. The P21 gene expression data described a very similar scenario seen in the P10 datasets: The three heterozygous genotypes displayed a graded degree and number of genes affected in the mutants relative to the P21 WT control (Table 1; Figure S6a–e in Additional file 8).

Next, we compared expression changes in the heterozygous mutants between P10 and P21 relative to P10 WT expression levels. In WT mice, 678 genes showed expression changes between P10 and P21 (FC ≥ 2 or ≤ −2, FDR ≤ 0.5), as shown in a heatmap (Figure S7a in Additional file 9). These age-dependent gene expression changes likely reflect retina terminal differentiation: GO analyses showed that the top 100 up-regulated genes were enriched for those important for developing mature photoreceptor structure and function, including visual perception, detection of light stimulus, photoreceptor outer segment formation and monovalent inorganic cation transport (Figure S7b in Additional file 9). In contrast, the down-regulated gene set was composed of genes that are important for neurogenesis during development, including system development, cell adhesion and regulation of cell proliferation (Figure S7b in Additional file 9). We next analyzed age-dependent gene expression changes in the *Crx* mutant lines, primarily focusing on *E168d2/+* and *E168d2neo/+* models, because of their distinct phenotype from WT mice. Unexpectedly, both mutants showed a trend of changes similar to WT mice for P21 up-regulated and down-regulated gene sets (Figure S7a, c in Additional file 9). In mutants, however, expression of many members of these two gene sets did not reach the WT level at P21 (Figure S7a, c in Additional file 9). As expected, this defect was more prominent in *E168d2/+* than *E168d2neo/+* mice. This suggests that the *E168d2/+* mutants continue to develop after P10 and recover some gene expression, but the degree of recovery is insufficient to achieve normal photoreceptor maturation. Interestingly, *R90W/+* also displayed slightly altered expression levels of many dynamically changed genes. These subtle differences may eventually contribute to the minor functional deficits observed for *R90W/+* at 6 months of age [21]. Taken together, these data suggest that *Crx* mutant mice fail both in repressing developmental genes and in activating genes required for photoreceptor maturation. These age-related gene expression changes support the morphological and functional observations that *Crx* mutant mice vary in their photoreceptor maturation rates.

### Down-regulated and up-regulated genes in *Crx* mutants show distinct epigenetic profiles in WT retinas

**Fig. 5:**
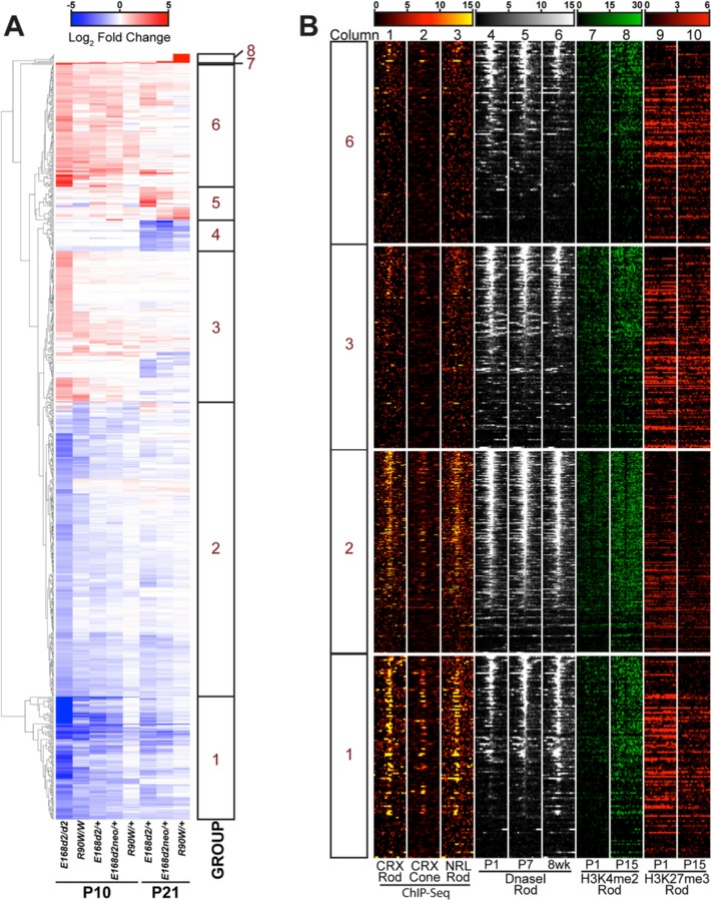
Hierarchical clustering and epigenetic data reveal groups of similarly regulated genes. **a** Hierarchical clustering analysis of all genes that showed significantly altered expression (FC ≥ 2 or ≤ −2, FDR ≤ 0.05) in at least one mutant genotype relative to age-matched WT expression. Expression levels in the indicated genotypes at the indicated ages are indicated by the *blue–red* heatmap. Eight groups of genes (indicated by the *bars* on the *right*) are clearly defined by the results (see Additional file 11 for designations). **b** Heatmaps showing the epigenetic landscape near the transcription start site (TSS) of genes in groups 1, 2, 3, and 6. Each row represents ±1 kb from the TSS (at *center* of panel) of individual genes contained within the groups as noted on the *left*; rows are ordered within each group by decreasing DNase I hypersensitivity (DHS) at the TSS in the 8-week dataset. *Columns 1–2*, CRX binding determined by ChIP-seq in adult WT and *Nrl−/−* retinas, respectively; *column 3*, NRL ChIP-seq in adult WT retinas; *columns 4–6*, DHS of WT retinas at three indicated ages; *columns 7–8*, H3K4me2 ChIP-seq in WT retinas at P1 and P15, *columns 9–10*, H3K27me3 ChIP-seq in WT retinas at P1 and P15. Quantification is presented in Additional file 13

To determine the modality of CRX’s regulation of differentially expressed genes, we further investigated their expression patterns and the epigenetic landscape of their proximal cis*-*regulatory regions in WT mice. We first used hierarchical cluster analysis on P10 and P21 datasets to find sets of genes that were similarly affected in all mutants. Figure 5a shows a heatmap representing log_2_ FC relative to age-matched WT samples for any gene that displayed significant change from WT (FC ≥ 2 or ≤ −2, FDR ≤ 0.05) in any single genotype; the data are arranged by hierarchical clustering (clustering branches are shown to the left of the heatmap). By visual inspection of clustered data, we further subdivided affected genes into eight groups based on similarity of altered expression patterns. These are designated as groups 1–8 (shown to the right of the heatmap; see Additional files 10 and 11 for lists and order of genes). Further analyses focused on groups 1, 2, 3 and 6, as these represented the largest and most consistent clusters. Visual inspection of biological replicate data also confirmed the consistency of the expression changes in these groups (Additional file 12). Group 1 genes were the most down-regulated genes across all genotypes. Group 2 genes were decreased compared with WT levels, but across the board were less affected than those in group 1. Groups 3 and 6 were composed of genes that were up-regulated in many of the genotypes. Group 6 genes generally were up-regulated to a greater extent.

#### The proximal *cis*-elements of down-regulated genes are enriched for CRX and NRL binding

To determine which of the above four major gene groups are directly regulated by CRX and its interacting rod-specific transcription factor NRL, we analyzed previously published CRX and NRL ChIP-seq data [15, 17] obtained from adult mouse retinas for all of the genes in these four clusters. Since CRX is expressed by both rods and cones, CRX ChIP-seq data from both WT (predominantly rod) and *Nrl−/−* (predominantly cone) retinas were included in our analyses. The WT NRL ChIP-seq data represent rod data, since NRL is not expressed in cones. The data are presented in heatmaps in Fig. 5b (columns 1–3), where each gene within the group is represented by a single line. The data within each column report the average read depth of the indicated experiment ±1 kb in 40 bp bins centered on the transcription start site (TSS). These results presented interesting contrasts between the various groups of genes: First, groups 1 and 2 displayed a significant amount of CRX binding in rods (column 1) and cones (column 2) and NRL binding in rods (column 3) around the TSS, while this binding was virtually absent in groups 3 and 6 (Fig. 5b, columns 1–3; Figure S9a–c in Additional file 13). This suggests that the down-regulated genes in groups 1 and 2 are enriched for direct CRX/NRL targets, but not the up-regulated genes in groups 3 and 6, consistent with the primary role of CRX and NRL in transactivation. Second, the more extensively down-regulated genes in group 1 showed more CRX (columns 1 and 2) and NRL (column 3) binding than the less-severely down-regulated genes in group 2 (Fig. 5b, columns 1–3; Figure S9a–c in Additional file 13), raising the possibility that group 1 genes are more dependent on CRX/NRL transactivation activity than group 2 genes. Third, comparison of CRX binding in rods (column 1) versus cones (column 2) for group 1 genes showed a high degree of CRX binding in both photoreceptor subtypes, suggesting that this group contains genes that are activated by CRX in both rods and cones. In contrast, group 2 genes showed CRX binding largely in rods, suggesting these genes are activated by CRX mainly in rods (Figure S9a, b in Additional file 13).

#### Down-regulated genes become more “open” during postnatal retinal development

To determine which of the four major groups undergo epigenetic landscape changes during photoreceptor development, we analyzed three different sets of previously published and publicly available epigenetic signature data (Fig. 5b, columns 4–10). Results are presented as heatmaps in a similar format as CRX and NRL ChIP-seq data.

We first analyzed retinal DNase I hypersensitivity (DHS) datasets at the age of P1, P7 and 8 weeks (Fig. 5b, columns 4–6) from the ENCODE project [22]. Enriched DHS is an indicator of ‘open’ chromatin and is a predictor of active transcription. Both down-regulated and up-regulated genes showed dynamic changes in their epigenetic landscapes with age. First, group 1 genes showed increases in DHS signal from P1 to 8 weeks of age. This pattern is not seen in group 2 genes, in which high DHS signal was largely stable across the ages tested. Group 3 and 6 genes, on the other hand, showed different patterns, in which DHS signal decreased from P1 to 8 weeks of age. Second, comparing across groups at 8 weeks of age, DHS data described groups 1 and 2 as having more ‘open’ chromatin (higher DHS signal) around their TSS than groups 3 and 6 (Fig. 5b, column 6). These data suggest that the chromatin of genes down-regulated in *Crx* mutants (groups 1 or 2) have ‘open’ chromatin in the adult WT retina and the most strongly down-regulated genes (group 1) are genes that develop a more ‘open’ chromatin conformation postnatally. In contrast, up-regulated genes (groups 3 and 6) tended to have ‘open’ chromatin in the early postnatal retina that became more ‘closed’ with age. To determine if these dynamic DHS changes represented specific events for particular groups, we included a random control group in our analyses. A random set of genes was chosen from the UCSC Table Browser ‘UCSC Genes’ list (see “Materials and methods”) to match the size of the largest group from the analysis (“random” group). As illustrated by Figure S9d–f in Additional file 13, the random group (grey lines) also showed a trend of reduction in DHS during postnatal retinal development. Importantly, when compared with random group genes, group 1 genes were less ‘open’ at P1 and P7 (Figure S9d, e in Additional file 13, blue line versus grey line) but more ‘open’ at 8 weeks (Figure S9f in Additional file 13, blue line versus grey line), verifying the trend visible by eye in the Fig. 5b heatmaps. These results were consistent with the observation that group 1 genes largely increased their expression from P2 to P21 during WT retinal development (Figure S9k in Additional file 13, based on published RNA-seq data by [24]). Interestingly, group 2 genes were more ‘open’ than random group genes at all ages tested (Figure S9d,f in Additional file 13, dashed blue line versus grey line), consistent with an overall modest increase in their expression from P2 to P21 in WT mice (Figure S9l in Additional file 13). In contrast, group 6 genes showed overall lower DHS than random group genes at all three ages analyzed, while group 3 genes showed similar DHS patterns as random group genes. Both group 3 and 6 genes normally showed no change or a slight decrease in expression from P2 to P21 (Figure S9m, n in Additional file 13). These data suggest the DHS changes identified in group 1 genes are dynamically regulated during retina development.

#### Down-regulated genes undergo histone modification changes during postnatal retinal development

Next, we analyzed these gene groups for the presence of active (H3K4me2; columns 7 and 8) and repressive (H3K27me3; columns 9 and 10) histone marks as determined by ChIP-seq in P1 and P15 WT retinas [23] within the 2-kb window centered on the TSS of each gene. Consistent with gaining a more ‘open’ chromatin configuration, group 1 genes gained the active mark H3K4me2 and lost the repressive mark H3K27me3 between P1 and P15 (Fig. 5b, columns 7–10; Figure S9g–j in Additional file 13, blue line versus grey line). Group 2 genes showed similar changes in histone marks (increase in H3K4me2 and decrease in H3K27me3) from P1 to P15. Group 2 genes also showed a higher level of the active mark H3K4me2 than random group genes at P1, while the level of the repressive mark H3K27me3 was similar to random at this age (Fig. 5b, columns 7–10; Figure S9g–j in Additional file 13, dashed blue line versus grey line). Overall, the data are consistent with a postnatal constitutively open chromatin configuration for group 2 genes as measured by DHS. Finally, groups 3 and 6 showed no difference relative to the random group genes in either H3K4me2 or H3K27me3 occupancy through retina development (Fig. 5b, columns 7–10; Figure S9g–j in Additional file 13, red lines versus grey lines), consistent with their overall constant low level of gene expression.

Together, the above analyses suggest that group 1 genes undergo dynamic epigenetic changes during retina maturation associated with their substantial transcriptional activation during postnatal photoreceptor development. The expression pattern of these genes correlated with CRX and NRL expression, and their regulatory regions were directly bound by CRX and NRL in WT mice. These results suggest that group 1 is enriched for genes that are inactive in precursor cells and actively turned on by key photoreceptor transcription factors during development. Thus, mutations in CRX have a profound negative impact on the expression of these genes. Group 2 genes were similar to group 1 in that their epigenetic landscape is supportive of high transcriptional levels in the WT retina and are likely regulated directly by CRX and NRL. Interestingly, these genes do not show the time-dependent chromatin ‘opening’, suggesting that CRX and NRL may not be necessary to initiate chromatin remodeling and could account for the fact that group 2 genes lose expression to a lesser degree than group 1 genes. Finally, group 3 and 6 data suggest that these genes undergo chromatin remodeling in the WT retina that results in a less permissive state. This is consistent with their low or even decreasing expression over time. However, this process is not directly controlled by CRX or NRL, as ChIP-seq binding of these two proteins was not observed in these groups.

### Up-regulated genes are characteristic of cone photoreceptors, likely resulting from de-repression in rods

**Fig. 6:**
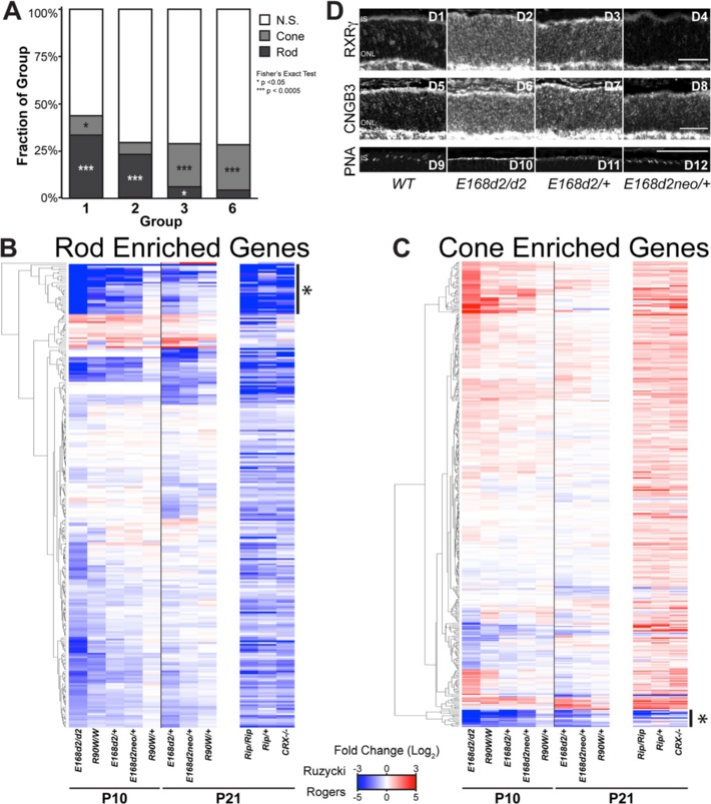
*Crx* mutants lose rod gene expression but up-regulate many phototransduction-unrelated cone gene transcripts. **a** Classification of genes within groups 1, 2, 3, and 6 as rod-enriched (*dark grey*), cone-enriched (*light grey*), or not specific to a particular cell type (*N.S.*, *white*) in the normal adult retina. Rod and cone-enriched genes are defined based on the comparison of published P21 WT to *Nrl−/−* RNA-seq data [24]. Rod: FC ≥ 2, FDR ≤ 0.05. Cone: FC ≤ -2, FDR ≤ 0.05. Fishers exact test, *p < 0.05, ***p < 0.0005. **b**, **c** Heatmaps depict hierarchical clustering of FC relative to WT for all rod-enriched (**b**) and cone-enriched (**c**) genes. Expression data from a published RNA-seq study describing the *Crx Rip/+*, *Rip/Rip*, and *Crx−/−* mice are also separately presented in the order determined by the aforementioned clustering. Regions noted in (**b**) and (**c**) with asterisks are presented in larger format in Additional file 14 and represent many down-regulated genes involved in phototransduction in rods and cones. **d** Immunohistochemical staining for RXRγ, CNGB3, and peanut agglutinin (*PNA*) in P10 WT and three Crx mutant retina sections. Scale bar = 100 μm. *IS* inner segment, *ONL* outer nuclear layer

Since CRX and CRL binding as well as epigenetic data implicate down-regulated genes as being active CRX targets in rods, we investigated if differentially expressed genes normally have rod or cone cell type-specific expression patterns. Using published data [24] to classify these genes as either rod- or cone-enriched (or non-specific), we found a significant overrepresentation of rod genes in groups 1 and 2, although a number of cone genes were also found in group 1 (Fig. 6a; Additional file 10). This was expected considering the mouse retina is rod-dominant and CRX acts as a transcription activator for genes critical for photoreceptor structure and function. Surprisingly, however, up-regulated groups 3 and 6 contained a significant enrichment of cone transcripts (Fig. 6a; Additional file 10).

To determine whether this up-regulation of cone gene expression is a general trend beyond group 3 and 6 genes, we expanded our analysis to all genes enriched in either rods or cones in adult mice. Furthermore, to ensure the results are applicable to other classes of *Crx* mutants, we also analyzed three previously published RNA-seq datasets from other *Crx* mutant models, *Crx−/−*, *Rip/+* and *Rip/Rip*. The results, presented as heatmaps (Fig. 6b, c), showed a broad switch in the global pattern of gene expression in all mutants, with loss of expression of a large set of rod genes (Fig. 6b) and increased expression of transcripts normally enriched in cones (Fig. 6c). There were exceptions: expression of a small number of cone genes (indicated with an asterisk at the bottom of Fig. 6c) was consistently decreased in all the *Crx* mutants. These represented key cone-specific phototransduction genes, including *Pde6h*, *Arr3*, *Opn1mw*, *Opn1sw*, *Gnat2*, and others (Figure S10b in Additional file 14). Their loss of expression was similar to rod-specific phototransduction components that also consistently decreased, including *Rho*, *Gnat1*, *Cngb1*, and *Pde6g* (Fig. 6b, marked by asterisk; Figure S10a in Additional file 14). Again, rod-enriched genes and cone-enriched genes displayed graded changes that reflected phenotype severity in our models (most severe in *E168d2/d2*, least severe in *R90W/+*). These results also place the *Rip* model (*Rip/+* and *Rip/Rip*) in line with other *Crx* models, demonstrating even more severe expression changes than *E168d2/d2*, although the RIP protein is thought to cause disease by a distinct molecular mechanism [24].

Because RNA-seq was performed on whole-retina samples, the down-regulation of rod genes and up-regulation of cone genes could represent a decrease in the number of rods and an increase in cones. However, previously published histology showed that, in fact, the proportions of these cell populations shift in the opposite direction, as the *E168d2/+* retina has a very severe and early depletion of cone photoreceptors, prior to any loss of rod photoreceptors [21]. In fact, all *Crx* mutant mouse models, including *Crx−/−* and *Rip/+*, show a similar trend of rapid cone loss followed by slower rod degeneration. This result was also unexpected considering the role of CRX in trans-activating cone as well as rod genes [21, 28]. This raised the possibility that the up-regulated cone genes were abnormally derepressed in the mutant rods. To further understand the molecular mechanism underlying mis-expression of cone genes in mutant rods, we assessed all rod- and cone-enriched transcription factors for expression changes in the mutants. Consistent with overall expression changes, we observed a general loss of rod transcription factors and enhanced expression of many cone-enriched transcription factors (Figure S11a, b in Additional file 15). However, the reduced expression of genes encoding rod-specific factors essential for maintaining rod cell fate, such as *Nrl* and *Nr2e3*, was rather minor or even absent in the heterozygous *E168d2 and R90W* mutants, unlike that reported for *Rip* mice [24]. Instead, these results suggest an unexpected role for CRX in rods to repress cone-enriched genes, including a number of cone-enriched transcription factors. To test whether CRX could play a direct role in repressing these cone-enriched transcription factors, we examined the CRX ChIP-seq data (Figure S11a–c in Additional file 15) for evidence supporting a direct interaction of CRX with up-regulated cone transcription factors in rod cells. Some up-regulated cone transcription factors showed low chromatin immunoprecipitation (ChIP) signal in WT retina, suggesting a potential repressive binding in rods, but the pattern was not consistent (Figure S11c in Additional file 15). Thus, loss of repression of cone genes in mutants is likely a secondary effect. In contrast, ~33 % of rod genes were bound by CRX, significantly enriched over random genes (~8 %). Furthermore, when compared with NRL ChIP-seq data, >73 % of CRX-bound rod genes were also bound by NRL, but no enrichment of NRL binding was observed for cone genes (data not shown), consistent with CRX’s interaction with NRL to activate the expression of rod genes, but not cone genes.

To verify that these cone transcripts are being de-repressed in mutant photoreceptors that normally would adopt a rod cell fate, we performed immunohistochemistry for several cone targets on retinal sections from three *E168d2* sublines and WT control mice at P10. The nuclear receptor RXRγ (retionoid X receptor gamma) is preferentially expressed by cones in P10 WT retina, and is up-regulated in most *Crx* mutants by RNA-seq and qRT-PCR [21] (Table S1a, b in Additional file 1; biological replicate RNA-seq raw mapped sequencing reads displayed in Figure S2a, b in Additional file 4). As shown in Fig. 6D (panels D1–D4), instead of the cone-expression pattern in normal retina (Fig. 6d, panel D1), RXRγ immunoreactivity was detected in most outer nuclear layer (ONL) cells of the homozygous and heterozygous *E168d2* mutant retinas (Fig. 6d, panels D2 and D3), with a perinuclear distribution similar to the pattern reported for rod transcription factors [8]. The ONL RXRγ staining appeared stronger and more widely spread in *E168d2/d2* than *E168d2/+* retinas. The enhanced RXRγ immunoreactivity was not seen in *E168d2neo/+* ONL where the rods were not disturbed. Immunostaining of the second cone marker, CNGB3 (cyclic nucleotide gated channel beta 3), displayed similar patterns of enhanced ONL expression in affected mutant rods (Fig. 6d, panels D5–D8). We next assessed peanut agglutinin (PNA) binding (Fig. 6d, panels D9–D12), which normally stains the cone sheath including cone outer segments and pedicles. Figure 6d shows that, despite the lack of cones and rod outer segments in *E168d2/d2* retinas, a strong and uniform PNA staining was seen across the outer margin of the entire retina (Fig. 6d, panel D6), suggesting that mutant rods adopt cone-like characteristics of their sheath. *E168d2neo/+* retinas did not show enhanced PNA binding at this age (Fig. 6d, panel D12), while *E168d2/+* showed an intermediate PNA staining that was higher than in WT but much lower than in *E168d2/d2* retinas. Taken together, all three selected cone markers showed enhanced expression in mutant rods in the order *E168d2/d2* > *E168d2/+* > *E168d2neo/+*, thus confirming RNA-seq findings of the impact of *Crx* mutations on rod cell fate.

**Fig. 7:**
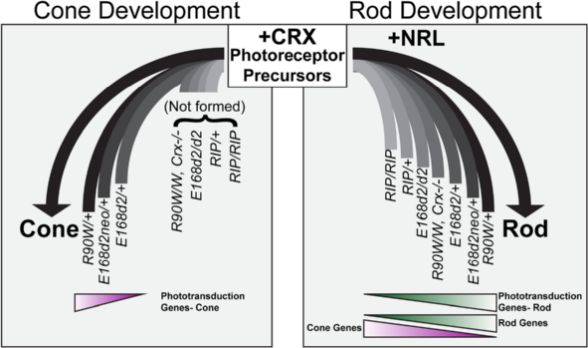
Model of how *Crx* mutation-caused gene expression changes affect rod and cone development. The *left panel* describes the formation of cones in a subset of the *Crx* mutants and variable levels of those cells’ expression of phototransduction genes. The *right panel* shows how development of rods in all models is related to their gene expression changes. It also emphasizes the novel findings that *Crx* mutant rods display a graded phenotype of both the decreased expression of proper rod genes, and the mis-expression of cone genes

In summary, our RNA-seq analyses identified graded expression changes of shared gene sets in seven available mouse models for *CRX*-associated disease. Using the data presented and referenced in this paper [2, 21, 24], these models can be ranked as illustrated in Fig. 7, with the lightest bars representing the model with most severely affected rod and cone gene expression. This order correlates with phenotype severity by morphological and electrophysiological standards, and with changes in gene expression in rods and cones. This correlation was seen not only for down-regulated rod and cone genes encoding phototransduction components, but also for those up- and down-regulated gene sets that highlight the partial rod to cone conversion of the developing photoreceptors. The schematic also predicts the level of photoreceptor identity and function in various models. Most importantly, the different gene expression changes between some phenotypically distinct *Crx* mutant models (such as *E168d2/+* versus *E168d2neo/+*) were rather modest, in contrast to their substantial impact on the disease phenotype.

### Testing the effect of gene expression changes on light-dependent degeneration

#### *E168d2neo*/+ but not *E168d2*/+ mice are sensitive to light damage

**Fig. 8:**
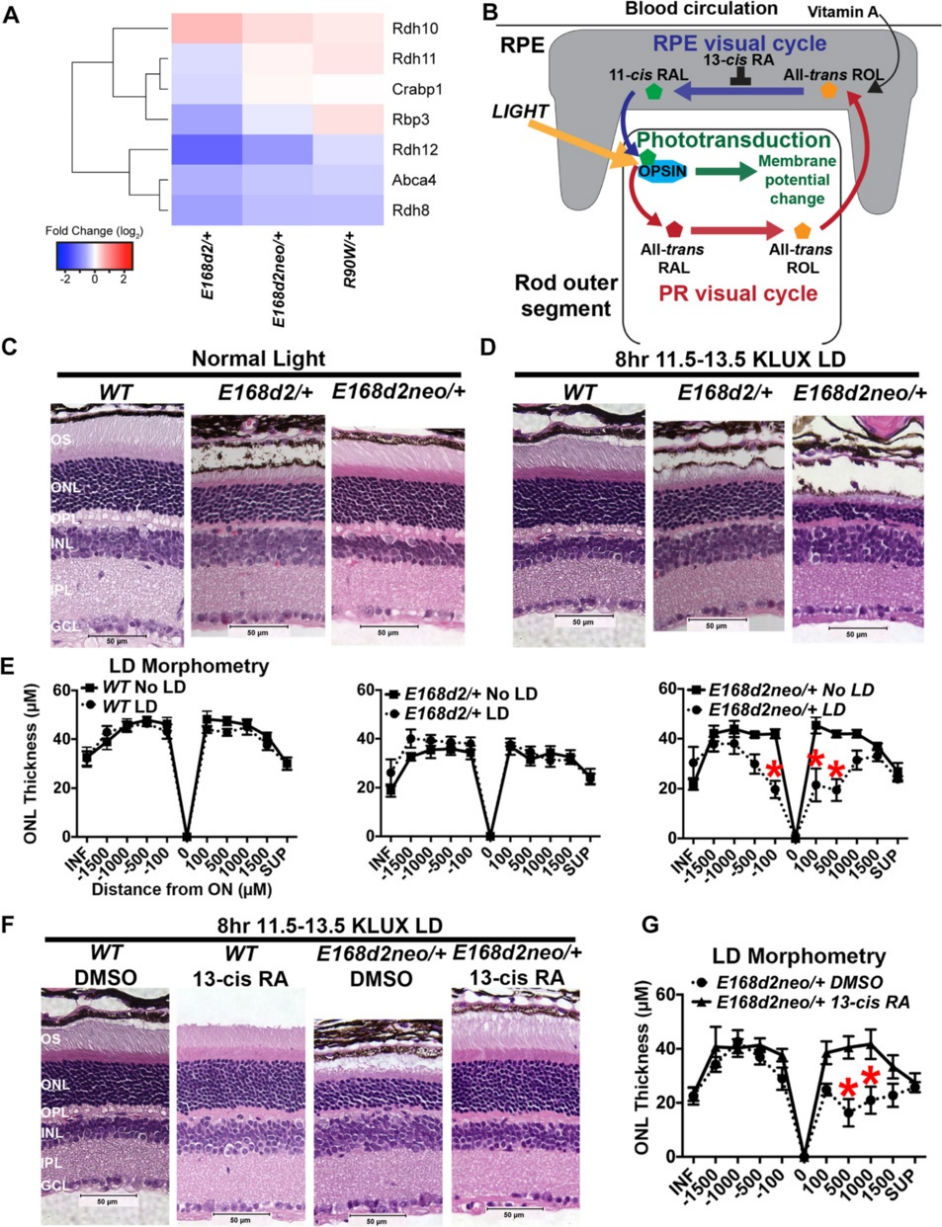
Light-dependent photoreceptor degeneration in *E168d2neo/+* mice. **a** Heatmap presents P21 *Crx* mutant FC relative to WT for genes involved in the photoreceptor visual cycle. **b** Model for the RPE visual cycle: visual chromophore [11-*cis*-retinal (*RAL*)] is generated in the retinal pigmented epithelium (*RPE*) and delivered to the photoreceptor; stimulation of 11-*cis* RAL bound to an opsin protein activates the phototransduction cascade leading to photoreceptor membrane hyperpolarization; this process isomerizes 11-*cis* RAL into all-*trans* RAL, which is then recycled back into 11-*cis*-RAL through a series of enzymatic steps, known as the visual cycle, in the photoreceptor and RPE. The drug 13-*cis* retinoic acid (*13-cis-RA*) blocks the synthesis of 11-*cis*-RAL, effectively reducing the burden on the retinoid cycle. *ROL* retinol. **c**, **d** Retinal morphology of 6-week-old mice with indicated genotypes under normal light conditions: 12 h room light–dark cycle (**c**) or light damage (*LD*) treatments (8 h 11.5–13.5 KLUX bright light followed by 7 days of normal light–dark cycles) (**d**). Retinal morphology was assessed by hematoxylin and eosin (H&E) staining of sagittal retinal sections through the optic nerve (ON). Images were taken in the central superior retina (~500 μm from the ON). **e** Morphometry quantification of ONL thickness for the samples presented in (**c**, **d**). Error bars represent standard error of mean (SE) from three or more biological replicates and significance was calculated using two-way ANOVA. Data points that significantly differ from the control (*p* ≤ 0.05) are marked by *red asterisks*. **f**, **g** Effect of 13-*cis* retinoic acid (*13-cis-RA*) pretreatment on LD in *E168d2neo/+* mutant mice: retinal morphology (**f**) and morphometry (**g**) at 7 days following LD where mice were pretreated with 13-*cis*-RA or dimethyl sulfoxide (*DMSO*) as control. Note the significant improvements in ONL thickness and photoreceptor outer segment (*OS*) length in the mice treated with 13-*cis*-RA. *GCL* ganglion cell layer, *INL* inner nuclear layer, *IPL* inner plexiform layer, *ONL* outer nuclear layer, *OPL* outer plexiform layer, *OS* outer segment. Scale bar = 50 μm

To validate the phenotypic significance of the small differences in gene expression between *E168d2/+* and *E168d2neo/+* mice (Fig. 4c), we analyzed expression of genes involved in the photoreceptor retinoid (visual) cycle that is responsible for the recycling of visual pigment chromophore 11-*cis*-retinal by RNA-seq (Fig. 8a; Figure S2a, b in Additional file 4) and qRT-PCR (Table S1c in Additional file 1). This discrete set of visual cycle genes expressed in photoreceptors also showed a similar pattern of expression changes across the mutant lines (Fig. 8a): *R90W/+* showed little or no changes relative to WT, while *E168d2/+* and *E168d2neo/+* showed reduction in gene expression levels. We validated the expression patterns of three visual cycle genes, *Rbp3*, *Rdh12* and *Abca4*, by qRT-PCR and results were consistent with RNA-seq findings (Table S1c in Additional file 1). We hypothesized that, as a result of these changes combined with slight shifts in expression levels of phototransduction genes (Fig. 4b), the two *E168d2* sublines may have different sensitivity to light-induced damage (LD). Exposing mice to high intensity light for an extended period of time puts stress on the retina and can lead to photoreceptor degeneration [29]. LD-related degeneration depends on three interconnected pathways (Fig. 8b): 1) the phototransduction pathway, 2) the visual cycle in the retinal pigment epithelium (RPE) and 3) the visual cycle in photoreceptor cells. Most mouse models with disruptions to components of phototransduction are insensitive to LD [30], while mice with an impaired visual cycle in photoreceptors generally have increased sensitivity to LD [31–35]. To test if the transcriptional defects in *E168d2/+* and *E168d2neo/+* mice alter their sensitivity to LD, we exposed 6-week-old WT, *E168d2/+* and *E168d2neo/+* mice to a high intensity light [12–13.5 kilolux (KLUX); ~10–20 times brighter than ambient light conditions] for 8 h. Following LD exposure, mice were kept in a 12 h ambient light/dark cycle for 7 days before retinal function and morphology were assessed. All the mice tested were backcrossed to the *C57BL/6 J* background for more than ten generations, which is resistant to LD [36]. As expected, WT mice were highly resistant to LD and displayed normal retinal morphology (Fig. 8d versus Fig. 8c, left panels), normal ONL thickness as determined by morphometry (Fig. 8e, left panel), and normal retinal function as measured by ERG (Figure S12a in Additional file 16, black dashed lines versus black solid lines) compared with WT controls that were not exposed to LD. *E168d2/+* mice exposed to LD did not show differences in retinal morphology (Fig. 8d versus Fig. 8c, middle panels) or ONL thickness (Fig. 8e, middle panel) and showed only minor ERG differences compared with normal light-exposed *E168d2*/+ controls (Figure S12a in Additional file 16, red dashed lines versus red solid lines). In contrast, *E168d2neo*/+ mice exposed to LD showed shortened outer segments and a loss of ONL nuclei, ~3–4 nuclei compared with ~10–12 nuclei in normal light *E168d2neo*/+ mice (Fig. 8d versus Fig. 8c, right panels). Morphometry revealed significant reduction in ONL thickness at the inferior −100 μm and superior 100 μm and 500 μm positions, with the thickness at 500 μm being most affected (~58 % reduced; Fig. 8e, right panel). Retinal function was also affected with dark-adapted a-waves, dark-adapted b-waves and light-adapted b-waves all showing reduced maximal response amplitudes compared with *E168d2neo*/+ controls kept in normal light (Figure S12a in Additional file 16, blue dashed lines versus blue solid lines). These data suggest that *E168d2neo*/+ mice are more susceptible to LD than either *E168d2*/+ or WT mice, providing further insight into the distinct pathobiology of *E168d2/+* and *E168d2neo/+* mice.

#### *E168d2neo*/+ sensitivity to light damage is linked to abnormal visual cycle in photoreceptors

To determine if the increased sensitivity to LD in *E168d2neo*/+ mice reflects changes in expression of visual cycle genes, mice were pre-treated with 13-*cis* retinoic acid (13-*cis*-RA; isotretinoin) before LD exposure. 13-*cis-*RA interferes with the regeneration of visual chromophore (11-*cis*-retinal) and is a strong antagonist of the RPE-driven phase of the visual cycle [37]. Inhibiting the visual cycle at the earliest stage reduces the production of toxic retinoid intermediates in the retinoid pathway during LD (Fig. 8b). This treatment strategy was previously shown to effectively ameliorate retinal degeneration in mouse models with impaired visual cycle [34, 35, 37]. Retinas of WT mice pretreated with 13-*cis*-RA and exposed to LD were morphologically indistinguishable from dimethyl sulfoxide (DMSO)-injected controls (Fig. 8f, two left panels). In contrast, retinas of *E168d2neo*/+ animals pretreated with 13-*cis*-RA were almost completely protected from the rapid degeneration observed in DMSO-injected *E168d2neo/+* controls (Fig. 8f, two right panels). Retinas of 13-*cis* RA-pretreated *E168d2neo*/+ mice revealed significant improvements in ONL thickness at the superior 500 μm and 1000 μm positions (Fig. 8g, right panel) compared with those that were DMSO treated. Retinal function was also better preserved in 13-*cis*-RA pretreated mice. Amplitudes of dark-adapted ERG a-wave, dark-adapted b-wave and light-adapted b-wave were all significantly restored in 13-*cis*-RA pretreated *E168d2neo*/+ mice compared with DMSO-injected controls (Figure S12b in Additional file 16, green lines versus blue dashed lines). To validate that LD sensitivity is linked to intrinsic defects in the visual (retinoid) pathway, we tested rod dark adaptation in WT and *E168d2neo*/+ mice. Following >90 % rhodopsin photobleach, the dark adaptation of *E168d2neo*/+ mouse rods was substantially delayed compared with that in WT mice (Figure S12c in Additional file 16, blue line versus black line). Pretreatment of *E168d2neo*/+ mice with 13-*cis*-RA strongly delayed rod dark adaptation further (Figure S12c in Additional file 16, red line versus blue line), indicating that 13-*cis*-RA effectively blocked regeneration of visual pigment by the RPE visual cycle. Together, these data suggest that the increased sensitivity of *E168d2neo*/+ mouse retinas to LD is linked to intrinsic defects in the photoreceptor visual cycle.

Finally, to determine if the degeneration of photoreceptors in *E168d2*/+ mice is at all affected by ambient light conditions, we reared *E168d2*/+ mice under either a normal 12 h light/dark cycle or in constant darkness. Retinal morphology of *E1682*/+ mice raised under either light condition was indistinguishable at 3 and 6 months of age (Additional file 17), suggesting that photoreceptor degeneration in *E168d2*/+ mice is independent of light conditions. These results illustrate that photoreceptor degeneration in *E168d2*/+ and *E168d2neo*/+ mice is mediated by discrete light-independent and light-dependent mechanisms, respectively.

## Discussion

### Gene expression in animal models for *CRX*-associated retinopathies

Our RNA-seq results for mutant *Crx E168d2* and *R90W* mouse retinas improve and expand upon previously published genomic expression microarray studies and qRT-PCR on these mouse lines [21]. The increased sensitivity of this platform allowed for the detection of more differentially expressed genes, especially for genes with low expression level, and also for the detailed analysis of the modest expression level differences between heterozygous animals. In combination with RNA-seq data from *Crx−/−* and *Rip* mice [24], a comprehensive dataset of retinal gene expression now exists for *Crx* mutant models with a range of phenotypes that reflect human disease. We have used these datasets to identify key changes in gene expression that correlate with disease severity.

Our results suggest that even relatively small changes in gene expression level can have a profound effect on the delicate balance of cellular pathways critical for photoreceptor function and survival, especially phototransduction and the visual cycle. Our RNA-seq analysis of retinal gene expression in mutant *Crx* mouse models by several methods indicates graded changes in photoreceptor gene expression at every level from overall expression patterns down to specific cellular pathways. First, homozygous mice were always more severely affected than their heterozygous counterparts (Additional file 5). Among homozygous and heterozygous mice, expression changes from least to most severe consistently followed the pattern *R90W < E168d2neo* < *E168d2*. These patterns were observed for both down-regulated and up-regulated genes. A high degree of overlap existed in the differentially expressed genes among models (Figs. 2 and 3; Additional file 8) and few genes displayed opposite expression patterns between models (Fig. 5). This suggests that few unique gene targets exist between models, and non-overlapping genes largely result from genes with similar trends in expression that simply do not pass the significance threshold of the in silico analysis. Graded changes were also observed for genes involved in retinal development; *Crx* mutants showed impairment in both the activation and repression of genes that shift expression patterns from P10–P21 (Additional file 9). Despite having collected mRNA from the whole retina, we were able to determine that the differentially expressed genes mostly occurred in rod and cone photoreceptors by using known patterns of cell type-specific retinal gene expression (Fig. 4a). This was consistent with CRX’s expression pattern and role in photoreceptor maturation. Within photoreceptor-specific pathways involved in light response, namely rod and cone phototransduction and the visual cycle, all *Crx* mutant models displayed graded down-regulation (Figs. 4b and 8a). Aside from these pathways, down-regulated genes tended to be rod genes, while up-regulated genes tended to be cone genes de-repressed in rods (Fig. 6; Additional file 15). We investigated genes that did not fall into either category (Additional file 10) but did not find any other major trends beyond this. Expansion of this analysis by assessing expression of all known rod or cone-enriched genes again showed a graded pattern of expression changes in these genes (Fig. 6b, c), including rod and cone-specific transcription factors (Figure S11a, b in Additional file 15). Re-analysis of previously published microarray data [21] showed the same trends of gene expression changes (data not shown). Finally, by analyzing RNA-seq results from two previously published animal models, *Crx−/−* and *Rip*, we were similarly able to detect graded expression changes in a common gene set (Fig. 6). In all, our analyses show graded changes in gene expression at several levels in *Crx* mutant mice for both down-regulated and up-regulated genes.

### Different modalities for down-regulated and up-regulated gene expression

While *Crx* mutant mice showed both down-regulated and up-regulated expression (Table 2), more genes were down-regulated in every mouse model tested. This is consistent with CRX’s established role as a transcriptional activator, though CRX does act as a repressor in certain contexts [38]. Utilizing available CRX [15] and NRL ChIP-seq [17] data sets and epigenetic data sets including DHS from the ENCODE project [22] and active (H3K4me2) and repressive (H3K27me3) histone marks [23], we identified patterns in the proximal cis-regulatory elements of differentially expressed genes. CRX and NRL binding were enriched at genes that were down-regulated in *Crx* mutant models, suggesting these genes are direct targets, while genes that were up-regulated were not enriched for CRX or NRL binding, suggesting these genes are indirect targets (Fig. 5). DHS data showed that a group of strongly down-regulated genes in mutant models shifted from ‘closed’ to ‘open’ chromatin from P1 to 8 weeks old in WT rods, suggesting these genes are normally activated during postnatal retinal development (Fig. 5; Figure S9d–f in Additional file 13). In contrast, the data described that a group of up-regulated genes became more ‘closed’, suggesting these genes are being repressed during postnatal development (Fig. 5b; Figure S9d–f in Additional file 13). The group of down-regulated genes also showed an increase in the active histone mark H3K4me2 and a decrease in H3K27me3 during postnatal development in WT retina (Fig. 5b; Figure S9g–j in Additional file 13), indicative of active chromatin remodeling. Changes in these histone marks were not observed for up-regulated genes, suggesting these genes are not subject to this type of active chromatin remodeling. Together, these results suggest that genes down-regulated in *Crx* mutant models are direct targets of CRX and NRL that normally are activated during postnatal development through chromatin remodeling, while up-regulated genes are indirect targets of CRX and NRL that are normally repressed during postnatal development through an unknown mechanism. CRX is known to recruit co-activators with positive chromatin remodeling capacity, such as CBP/p300 and the STAGA complex [39, 40]. Thus, CRX and its interacting co-activators may play an essential role in active chromatin remodeling during development for the direct target genes represented by the down-regulated groups in *Crx* mutants. Future profiling of epigenetic changes in these *Crx* mutant models will provide additional support for this possibility.

### *Crx* mutations affect rod and cone development and transcriptional integrity

The graded expression changes in *Crx* mutant models have profound effects on the development of their rods and cones as illustrated in Fig. 7. Homozygous *Crx−/−*, *E168d2*, *R90W*, *Rip* and *Rip*/+ mice do not form physiologically functional photoreceptors due to a strong reduction in both rod and cone phototransduction gene expression. Other models show gradations in both retinal function and phototransduction gene expression. In all models studied, the development and survival of cones are affected earlier and more severely than rods, implicating CRX in the terminal differentiation of cones. Interestingly, in WT rods, CRX and NRL appear to mediate both the activation of rod-specific genes and the repression of cone-specific genes. The dual reduction in rod-gene expression and de-repression of cone genes in *Crx* mutant rods coincides with rods adapting more cone-like properties, including less condensed chromatin and shorter outer segments [21, 24]. In *Rip*/+ mice, this shift was found to coincide with loss of NRL expression by P21, indicating a complete loss of rod cellular identity. The loss of NRL expression does not occur in *E168d2*/+ mice by this age, but de-repression of cone genes in rods is still observed. While E168d2 protein does not affect the function of NRL in vitro [21], its effect on NRL in vivo is uncharacterized. Our results provide evidence that even though RIP and E168d2 mutant proteins act through different pathological mechanisms, the resulting pathologies arise from scaled changes in similar sets of genes.

### Gene expression dictates phenotypic thresholds

The animal models used in this study have wide-ranging phenotypes that match those observed in human patients. The magnitude of overall gene expression changes correlated with phenotype severity in every model tested, but not in a linear manner. Instead, there are thresholds in gene expression that determine phenotypic presentation. An example of this threshold effect is evident in the *E168d2/+* and *E168d2neo/+* mice. These mice have drastically different phenotypes but have only small differences in gene expression. As we have previously shown, the *neomycin* cassette retained in *E168d2neo/+* mice suppresses an accumulation of the mutant transcript by an unknown mechanism [21]. qRT-PCR results (Table S1a in Additional file 1) of the same biological samples used for RNA-seq confirm that the WT allele showed no compensation and maintained approximately 50 % of its normal expression. In contrast, the results of RNA-seq (Additional file 18) and qRT-PCR (Tables S1a, b in Additional file 1), combined with previously published western blots and immunohistochemistry [21], all support a significant overexpression of mutant *Crx* mRNA and protein in *E168d2/+* mice. This mutant allele-specific overexpression was not evident in *E168d2neo/+* mutants. This difference in expression of the dominant negative form of CRX resulted in very small difference in FC of down-regulated genes (median of −1.8 in *E168d2*/+ compared with −1.7 in *E168d2neo*/+ mice; Fig. 3f, g). Despite this slight difference, *E168d2*/+ mice had much more severe deficits in retinal function and photoreceptor degeneration. Under normal light conditions, *E168d2neo*/+ mice had little photoreceptor degeneration in early adulthood. However, *E168d2neo*/+ rods were highly sensitive to light damage while WT and *E168d2*/+ rods were largely resistant (Fig. 8). Targeting the visual cycle blocked sensitivity of *E168d2neo*/+ photoreceptors to LD, implicating this pathway in the degeneration phenotype. In contrast, the degeneration of *E168d2*/+ photoreceptors was independent of light and the visual cycle. Sensitivity to LD requires phototransduction and is mediated by the visual cycle, both of which are affected in the *E168d2* and *E168d2neo* mouse models. However, these results suggest that the balance in function between these two pathways could be critical for determining sensitivity to damaging light. Our data support the conclusion that phototransduction is too impaired and outer segment structural changes are already too severe in *E168d2*/+ mice to allow for further light-mediated degeneration, while *E168d2neo*/+ photoreceptors largely preserve their structure and substantial levels of phototransduction components to reveal visual cycle defects under intense light. These results highlight that even minor tuning differences in photoreceptor gene expression have a dramatic effect on the mechanisms of disease pathology. These findings could have significant clinical importance as patients with *CRX*-associated retinopathies might have different responses to environmental factors like damaging light levels, which would affect their clinical outcome. These results also provide clues for potential therapeutic intervention to target the visual cycle in patients with late-onset *CRX-*associated retinopathy.

## Conclusions

Linking genotype to phenotype for *CRX* retinopathies remains imprecise [41]. Genomic assessment of retinal gene expression suggests that the range of phenotypes of *Crx* mutant mouse models are driven by graded changes in photoreceptor gene expression. Since CRX has such wide-ranging function in photoreceptor transcription, mutations that sometimes just slightly alter the expression of many genes can have a profound effect on the resultant phenotype. We relate retinal gene expression to phenotypic thresholds in rod and cone photoreceptor development, cellular integrity, function and degeneration in several *Crx* mutant mouse models. We have utilized publicly available genomic datasets to gain insight into cell type-specific expression changes and the different modalities for gene down-regulation and up-regulation in these mice. We have demonstrated how slight differences in gene expression can alter retinal susceptibility to light-dependent degeneration in *E168d2*/+ and *E168d2neo*/+ mice. These findings provide evidence that *CRX* retinopathies stem from graded changes in photoreceptor gene expression, which could significantly contribute to phenotypic variability.

## Materials and methods

### Mice

Mice were housed in a barrier facility operated and maintained by the Division of Comparative Medicine of Washington University School of Medicine. All mice used in this study were backcrossed to *C57BL6/J* mice obtained from Jackson Laboratories (Bar Harbor, ME, stock number 000664) for at least ten generations. Each line was genotyped for common *rd1*, *rd8*, and *Rpe65* variants that affect retinal structure and function; all lines were negative for *rd1* and *rd8* and contain the *C57BL/6 J* RPE65^450M^ isoform. The *Crx* mutant lines *E168d2*, *E168d2neo* and *R90W* and their genotyping procedures were published previously [21].

All procedures involving mice were approved by the Animal Studies Committee of Washington University in St Louis (IACUC 20120246, expiration date 18 January 2016). Experiments were carried out in strict accordance with recommendations in the Guide for the Care and Use of Laboratory Animals of the National Institutes of Health (Bethesda, MD, USA), the Washington University Policy on the Use of Animals in Research; and the Guidelines for the Use of Animals in Visual Research of the Association for Research in Ophthalmology and Visual Science [41].

### RNA collection and library preparation

For each genotype and time point: three biological replicates were analyzed, and each replicate consists of four retinas from one male and one female mouse. P21 WT samples represent a randomized sampling of WT littermates of *Crx* mutants. Retinas were immediately processed for RNA using the PerfectPure RNA tissue kit (5 Prime). The quantity and quality of the RNA was assayed using a Bioanalyzer (Agilent). Samples with a minimum RNA integrity number (RIN) score of 8.0 were then selected for library construction. Library construction was performed by the Genome Technology Access Center (Washington University in St Louis). mRNA was isolated by poly(A) selection using Oligo-dT beads (mRNA Direct Kit, Ambion) and then fragmented by incubation at 94 °C for 2.5 min in an alkaline buffer (40 mM Tris acetate pH 8.2, 100 mM potassium acetate, 30 mM magnesium acetate) and reverse transcribed using Super Script III enzyme (Invitrogen) and random hexamers to yield double-stranded cDNA. The cDNA was blunt ended using Klenow DNA polymerase, followed by addition of an A base and ligation of Illumina sequencing adapters to the 3’ ends. All enzymes, including DNA polymerase, Klenow DNA polymerase, Klenow exo-, RnaseH, T4 DNA polymerase, and T4 polynucleotide kinase were purchased from New England BioLabs. Ligated fragments were amplified for 12 cycles using primers incorporating the unique index tags before being sequenced on the Illumina Hi-Seq 2000 using single reads extending 42 bases.

### RNA sequencing data analysis

We aligned 1 × 42 bp reads to the mouse genome (version mm9) with the sequence aligner TopHat2 (v.2.0.5) [42] using the following parameters: −a 5 -m 1 -i 10 -I 500000 -r 100 –p 4 --microexon-search –no-coverage-search -x 20 --segment-length 25. Dependencies included Bowtie (v.0.12.8) [43] and Samtools (v.0.1.18) [44]. Bedgraph files were generated using BEDTools (v.2.23.0) [45] and visualized using IGV. The HTSeq package (v.0.6.1p1) [46] was used to assign aligned reads to the gene annotation reference track (UCSC Genes Track, UCSC Table Browser, NCBI37/mm9, accessed 16 July 2014). This generated a raw read count per gene which was used in EdgeR [26] for detecting differentially expressed genes. For each of the genotype comparisons, genes that did not pass the filter criteria of CPM ≥ 5 in all replicates of at least one comparison group were removed prior to the analysis. Filtered count data were normalized by the EdgeR default normalization method, TMM, and differential expression analysis for each of the comparison groups was performed by the exact test. *P* values were subjected to Bonferroni and Hochberg multiple testing correction to include FDR. Downstream analysis was performed using custom Perl and R scripts. PCA was performed using the princomp function in the stats R package (v.3.1.1) [47]. Gene expression heatmaps were generated using the heatmap.2 function of the gplots R package (v.2.16.0) [48]. As several heatmaps include too many genes to properly label each row, we have listed the genes included in each heatmap in the order in which they were presented in Additional file 11.

Raw RNA-seq data were also obtained from previously published work: Gene Expression Omnibus (GEO) accession [GEO:GSE52006] [24]. These data were aligned also using TopHat and analyzed as described above using HTSeq and EdgeR.

### Epigenetic data analysis

Data were obtained from GEO under accession numbers [GEO:GSE20012] [15], [GEO:GSE38500] [23], and [GEO:GSM1014198, GSM1014175, GSM1014188] [22]. NRL ChIP seq [17] was downloaded from the NEI Data Share website, accessible through [49]. Data were analyzed based on the mouse reference genome (NCBI37/mm9) and UCSC known gene table. Heatmaps and line graphs depicting epigenetic data were generated using the UCSC defined TSS for each gene and the UNIX software package HOMER (v.4.7) [50]. Heatmaps were generated by importing the HOMER generated counts back into R, ordering by adult DNase 1 level at the TSS, and visualized by the heatmap.2 function in the gplots R package.

### GO analysis

GO analysis [51] was performed using the online g:Profiler tool [52]. Terms were filtered to only include the best per parent term.

### LD and 13-*cis*-RA treatment

For LD analysis, mutant mice and littermate controls were dark adapted overnight. Eyes were dilated with 1 % cyclogyl and 2.5 % phenylephrine hydrochloride. Mice were then placed in darkness for 30 min before LD. LD was performed in a temperature-controlled rat cage with the top removed and lined with reflective material to provide even illumination. Exposure was performed using white fluorescent light and the intensity was measured using a lux meter. Light intensity was 11–13.5 KLUX in all experiments. Mice were placed into cages containing moistened food pellets, and each animal received damaging light for 8 h. Mouse pupils were re-dilated and the cage position was rotated every 2 h.

For 13-*cis*-RA treatments, 13-*cis*-RA (isotretinoin; Sigma-Aldrich, R3255) was reconstituted in DMSO to a concentration of 0.013 mg/μl. 13-*cis*-RA (40 mg/kg) or an equal volume of DMSO vehicle control was injected into the mouse's interperitoneal cavity 12 h before LD and mice were dark-adapted overnight. Mice were re-dosed with 40 mg/kg 13-*cis*-RA 30 min before LD.

Following all LD experiments, mice were returned to a 12 h light–dark cycle under normal ambient light conditions (~100 LUX) for 7 days. After 7 days, retinal function was tested by ERG. The peak amplitude responses for rod-driven dark-adapted a-waves, rod ON bipolar cell-driven dark-adapted b-waves and cone bipolar cell-driven light-adapted b-waves were measured for a series of increasing light intensities as described below. Mice were then sacrificed and eyes were collected for retinal histology assessment (see below). Images were taken in the central superior retina (~500 μm from the optic nerve), which is the area of the retina that is most strongly affected by LD [53].

### Histology, immunohistochemistry and microscopy

Histology analyses of hemotoxylin and eosin (H&E)-stained paraffin-embedded retinal sections were performed as described previously [21]. For ONL morphometry, 20× composites of whole retinal sagittal sections stained with H&E were analyzed using ImageJ software [54]. The distance from the optic nerve was determined by drawing a curved line along the outer limiting membrane. The ONL thickness was measured at 100 μm, 500 μm, 1000 μm, and 1500 μm from the optic nerve and 200 μm from the peripheral edge on both the superior and inferior retina. Results are presented by a ‘spider graph’. The between-group differences in ONL thickness were determined by testing overall genotype–distance interactions (*p* < 0.05, n ≥ 3) of each treatment. Statistical comparison of data at each distance was performed using two-way ANOVA for repeated measurement data, followed by a post hoc test to adjust *p* value for multiple comparisons between each genotype and the WT control group SAS 9.3 (SAS Institutes, Cary, NC, USA).

Fluorescence immunohistochemistry was performed on frozen retinal sections. Eyes were enucleated and fixed for 30 min in 4 % paraformaldehyde on ice. Eyes were washed in phosphate-buffered saline (PBS) and left overnight in 30 % sucrose in PBS at 4 °C. A 50 % volume of O.C.T. (Tissue-Tek, optimum cutting temperature formulation) was added and rocked at room temperature for 1 h. Eyes were then transferred to 100 % OCT for 1 h before frozen in OCT on dry ice. Blocks were cut onto polylysine (Thermo Scientific) slides and stored at −80 °C. For staining, slides were removed and allowed to dry for 30 min before 10 min 4 % paraformaldehyde fixation. Slides were washed (2 × 5 min) with PBS and blocked at room temperature with 5 % nonfat dry milk/2 % bovine serum albumin (BSA)/10 % normal goat serum for 2 h. Slides were washed (3 × 5 min) with PBS before overnight incubation with primary antibody at 4 °C. Antibodies were diluted as below in PBS with 1 % BSA and 0.5 % Triton X-100. Slides were washed (3 × 5 min) in PBS. Secondary antibodies (and PNA antibody) were diluted in the same buffer as primaries, applied to the slides, and incubated for 1 h at room temperature, and washed again (3 × 5 min) with PBS before the application of coverslip.

Primary antibodies and dilutions used are as follows: rabbit anti-RXRγ (1:500, Santa Cruz Biotechnology, sc-555), rabbit anti-CNGB3 (1:500, from Xi-Qin Ding, University of Oklahoma), PNA conjugated to rhodamine (1:500, Vector Labs). Secondary antibodies (1:400) were goat anti-rabbit or mouse IgG antibodies coupled to Alexa Fluor A488 and rhodamine 568 (Invitrogen). All slides were counterstained with hard-set mounting medium with DAPI (Vectashield).

Brightfield and fluorescent imaging was performed using either an Olympus BX51 microscope and Spot RT3 Cooled Color Digital camera (Diagnostic instruments Inc.) or a Leica DM5500B microscope and Leica DFC365FX camera (Leica Microsystems, Buffalo Grove, IL).

### Electroretinography

Mice were dark-adapted overnight and anesthetized with intraperitoneal injection of a mixture of ketamine (80 mg/kg) and xylazine (15 mg/kg). Pupils were dilated with 1 % atropine sulfate. A passive-heating pad maintained animal’s body temperature at 37 °C. Scotopic ERG responses were measured from both eyes using corneal platinum-ring electrodes held in place by a drop of Gonak solution. Full-field ERGs were recorded with the UTAS-E 3000 system (LKC Technologies, Inc.), using Ganzfeld-derived test stimuli of calibrated white light intensity. The amplitude of ERG a-wave was determined from the baseline to the primary negative peak of the photoresponse. The amplitude of ERG b-wave was measured from the a-wave peak to the maximum of the secondary positive peak. Photopic ERG recordings were performed under room illumination (~25 cd · s m^−2^) after 5–10 min of light adaptation.

The rate of rod dark adaptation was determined from the recovery of maximal ERG a-wave amplitude after a 30-s exposure to light bleaching of >90 % of rhodopsin (delivered by 520 nm LEDs focused at the surface of mouse eye cornea and producing ~2.5 × 10^8^ photons μm^−2^ s^−1^), using test flashes of ~ 470 cd · s m^−2^ at indicated times after the bleach. In these experiments, mice were re-anesthetized subcutaneously every 30–40 min with a smaller dose of ketamine (~50 % of the initial dose). In addition, at the same time a drop of 1 % atropine sulfate was added to the eye surface to keep pupils dilated, and a 1:1 mixture of PBS and Gonak solutions was applied to the eyes to protect them from drying and maintain electrode contacts during extended recording sessions.

### Quantitative RT-PCR

RNA used for assays included RNA used for Illumina RNAseq library preparation and additional biological replicates. For each genotype, n ≥ 3 biological replicates, each replicate consisting of four retinas from one male and one female mouse, were collected. RNA isolation, cDNA preparation, and qRT-PCR reactions were performed as previously described [21]. Briefly, retina tissue was immediately processed for RNA using the PerfectPure RNA tissue kit (5 Prime) and quantified. cDNA was synthesized from 1 μg of RNA using the Transcriptor First Strand cDNA Synthesis kit (Roche Applied Science). A 10 μl qRT-PCR reaction mixture containing 1× EvaGreen with Low Rox reaction mix (BioRad), 1 μM primer mix, and diluted cDNA was prepared and run on a BioRad CFX thermocycler in triplicate. Data were analyzed using QBase software (Biogazelle). Relative gene expression was normalized to *Ubb* and *Tuba1b* and FC from WT was determined. Kruskal-Wallis and Dunn's multiple comparison tests were used to determine significant FC differences from WT (*p* < 0.05). Primer sets were designed using MacVector software and synthesized by IDT DNA technologies (Additional file 19).

### Data availability

Raw RNA seq data and primary analysis are available from GEO under accession [GEO:GSE65506].

## Additional files

Additional file 1: Table S1. Compiled comparison of RNAseq with new and previously published qPCR data. (XLSX 14 kb) Additional file 2: Table S2. Compiled list of read number for each library. (XLSX 47 kb) Additional file 3: Figure S1. P10 and P21 biological replicates clustered by principal component analysis (PCA) show expected distribution based on functional and morphological phenotypes. PC1 and PC2 represent greater than 98 % of the variance of the data in both analyses. (TIFF 24711 kb) Additional file 4: Figure S2. P10 (**a**) and P21 (**b**) browser shots of reads per million (RPM) normalized mapped read count for selected genes for each biological replicate show reproducibility of sequencing results between samples. (ZIP 312 kb) Additional file 5: Figure S3. Homozygous and heterozygous mutants show largely overlapping datasets. Venn diagrams comparing significantly affected transcripts (FC ≥ 2 or ≤ -2, FDR ≤ 0.05 relative to WT) in the indicated heterozygous and homozygous *Crx* mutant retinas. (TIFF 14588 kb) Additional file 6: Figure S4.
*Crx* mutants show little change to other non-photoreceptor phototransduction components. Heatmap depicts P10 FC relative to WT for genes involved in phototransduction enriched in non-photoreceptor retinal cell types in the heterozygous mutants. (TIFF 14588 kb) Additional file 7: Figure S5. Heatmap showing the Z-scores comparing three biological replicates of genes determined to be enriched in either *E168d2/+* (**a**) or *E168d2neo/+* (**b**) retinas. Genes are arranged in rows, clustering dendrogram is shown on the left. Patterns of expression across biological replicates are consistent, supporting the statistical significance of the difference calculated by EdgeR RNA-seq analysis. (TIFF 14588 kb) Additional file 8: Figure S6.
*Crx* mutants show graded changes in gene expression of overlapping gene sets at P21. **a**–**c** Log_2_ CPM from P21 heterozygous *E168d2/+* (**a**), *E168d2neo/+* (**b**), and *R90W/+* (**c**) mice (y-axes) are compared with log2 CPM from age-matched WT *C57Bl/6 J* (x-axes) mice. White letters highlight prototypical photoreceptor transcripts. White diagonal lines represent ±2 FC. **d**, **e** Venn diagrams illustrate the numbers of overlapping and distinct significantly affected genes (FC ≥ 2 or ≤ -2, FDR ≤ 0.05). (TIFF 14588 kb) Additional file 9: Figure S7.
*Crx* mutants do not abandon the developmental program, but many genes fail to reach proper expression levels. **a** Heatmap, ordered by the magnitude of expression change between P10 and P21 in WT retina, depicts FC compared with P10 WT to analyze developmental dynamics. **b** Top GO terms from analysis of top 100 genes that are down- (*left panel*) and up- (*right panel*) regulated normally during late postnatal retinal development (P21 WT versus P10 WT; FC ≤ −2 or ≥ 2, FDR ≤ 0.05). **c** Violin and boxplots quantifying median FC from P10 WT of each mutant. (TIFF 14588 kb) Additional file 10: Table S3. Compiled table of results from GO analysis on rod- or cone-enriched genes from Fig. 6a. (XLSX 50 kb) Additional file 11: Table S4. Compiled ordered lists of heatmap data presented in the manuscript. (XLSX 148 kb) Additional file 12: Figure S8. Groups 1, 2, 3, and 6 genes (defined in Fig. 5) presented as a heatmap with calculated FC values for each biological replicate compared with the mean age-appropriate WT value. All genes are ordered exactly as presented in Fig. 5. (TIFF 14588 kb) Additional file 13: Figure S9. Quantification of normal epigenetic marks near genes in groups 1, 2, 3, and 6 distinguishes up- and down-regulated genes in *Crx* mutants. Control set of equal sized random group of mm9 genes used as background control (*grey* in all). **a**–**c** Quantification of CRX ChIP-seq [15] in rod-dominant WT retina (**a**) and cone-dominant *Nrl−/−* retina (**b**), and NRL ChIP-seq [17] in WT retina (**c**). **d**–**f** DNase I hypersensitivity plots from WT retina at the three indicated ages [22]. **g**, **h** H3K4me2 ChIP-seq of WT retina at the two ages [23]. **i**, **j** H3K27me3 ChIP-seq of WT retina at the two ages. All represent normalized mean read depth of each library centered on the TSS of genes within groups 1, 2, 3, and 6. **k**–**n** Analysis of WT expression of each gene and average change in expression (*blue line*) from the age of P2 to P21 (raw data in [24]), presented as log_2_ CPM. (TIFF 14576 kb) Additional file 14: Figure S10. Expanded view of areas marked with asterisks in Fig. 6. All data and presentation exactly as described previously. (TIFF 14588 kb) Additional file 15: Figure S11. Changes in rod- and cone-enriched transcription factors are consistent with general patterns of rod and cone gene expression in *Crx* mutants. **a**, **b** Heatmap describes FC relative to WT in each *Crx* mutant for rod- (**a**) and cone-enriched (**b**) genes annotated as transcription factors (raw data in [24]; WT versus *Nrl−/−* RNA-seq FC ≤ −2 or ≥2 and FDR ≤ 0.05; GO:0003700). ChIP-seq data are presented for 4-kb windows surrounding the TSS for each gene for CRX (in rods and cones) and NRL (in rods). **c** Analysis detailing percent of rod-enriched, cone-enriched, or random set of genes with CRX ChIP-seq peak within 2 kb of TSS in WT retina. Note the significant enrichment of CRX binding in the rod set over cone and random gene sets. (TIFF 14588 kb) Additional file 16: Figure S12. Retinal function is affected by light damage (LD) in *E168d2neo/+* but not *E168d2/+* mutant mice. **a** Intensity-response plots for dark-adapted ERG a-wave, dark-adapted b-wave and light-adapted b-wave from mice with the indicated genotypes, with or without LD treatment. **b** Intensity-response plots for ERG responses following LD for mice pretreated with either 13-*cis*-RA or DMSO. **c** Rod dark adaptation measured by recovery of maximal rod ERG a-wave following >90 % rhodopsin photobleach for WT and *E168d2neo/+* mice, with and without drug treatment. Error bars represent standard error of mean (n ≥ 3). Asterisks mark data points of significant difference (*p* ≤0.05) from the control as determined by two-way ANOVA (see "Materials and methods"). (TIFF 3018 kb) Additional file 17: Figure S13.
*E168d2/+* photoreceptor degeneration is light-independent. Retinal morphology of *E168d2/+* mice raised under 12 h light–dark cycle (normal light) or constant darkness (dark-reared) for 3 or 6 months. Note that the dark rearing did not improve ONL thinning in the mutant retina. *GCL* ganglion cell layer, *INL* inner nuclear layer, *IPL* inner plexiform layer, *OS* outer segment, *ONL* outer nuclear layer, *OPL* outer plexiform layer. Scale bar = 50 μM. (TIFF 3603 kb) Additional file 18: Figure S14. Increased *Crx* expression in mutant lines at P10. **a** RNA-seq derived raw CPM values for each biological replicate (*red circles*) and mean (*black bar*). Top panel of spreadsheet lists the EdgeR calculated FC and FDR for each genotype relative to WT control. Asterisks denote samples where FDR < 0.05. **b** qRT-PCR analysis of transcript levels of WT and mutant *Crx* alleles in each genotype. Note the same trend of *Crx* expression changes in these mutants detected by both methods. (TIFF 14588 kb) Additional file 19: Table S5. List of primers used for qRT-PCR completed for this manuscript. (XLSX 54 kb)

## Abbreviations

bp: base pair
BSA: bovine serum albumin
ChIP: chromatin immunoprecipitation
CNGB: cyclic nucleotide gated channel beta
CoRD: cone-rod dystrophy
CPM: counts per million (of sequence reads)
CRX: Cone-rod homeobox protein
DHS: DNase I hypersensitivity
DMSO: dimethyl sulfoxide
ERG: electroretinography
FC: fold change
FDR: false discovery rate
GEO: Gene Expression Omnibus
GO: Gene Ontology
H&E: hematoxylin and eosin
IGV: Integrated Genomics Viewer
KLUX: kilolux
LCA: Leber congenital amaurosis
LD: light damage
NRL: Neuroretina leucine-zipper protein
ONL: outer nuclear layer
P: postnatal day
PBS: phosphate-buffered saline
PCA: principal component analysis
PNA: peanut agglutinin
qRT-PCR: quantitative real time PCR
RA: retinoic acid
RNA-seq: mRNA sequencing
RPE: retinal pigment epithelium
TSS: transcription start site
UCSC: University of California, Santa Cruz
WT: wild type (*C57BL/6 J*)
