# Supplementary figures and images for: Graded gene expression changes determine phenotype severity in mouse models of CRX-associated retinopathies
Source: Genome Biol. 2015 Sep 1;16(1):171. doi: 10.1186/s13059-015-0732-z (PMC4556057; doi:10.1186/s13059-015-0732-z)

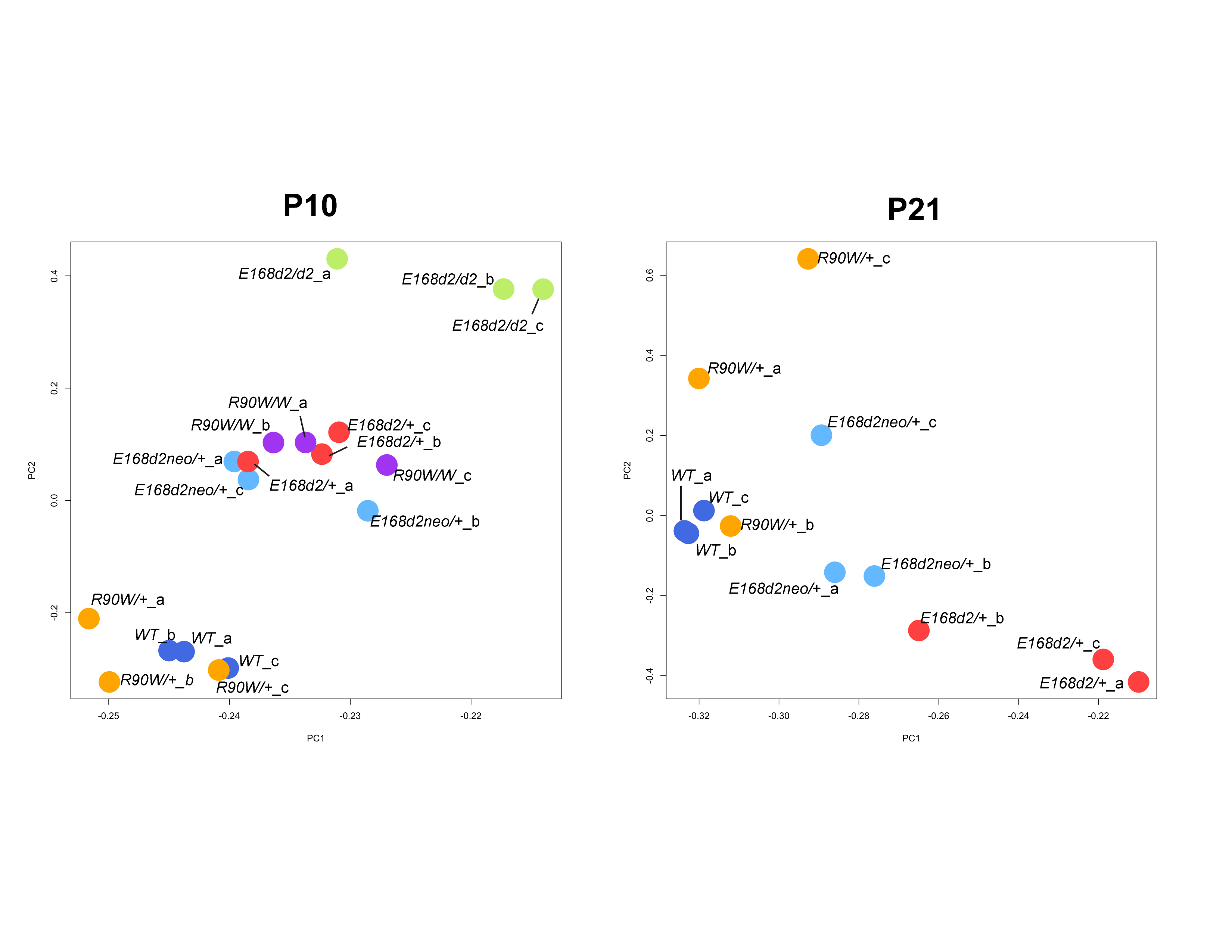

Supplement: Additional file 3: Figure S1. — P10 and P21 biological replicates clustered by principal component analysis (PCA) show expected distribution based on functional and morphological phenotypes. PC1 and PC2 represent greater than 98 % of the variance of the data in both analyses. (TIFF 24711 kb) [file 13059_2015_732_MOESM3_ESM.tif]

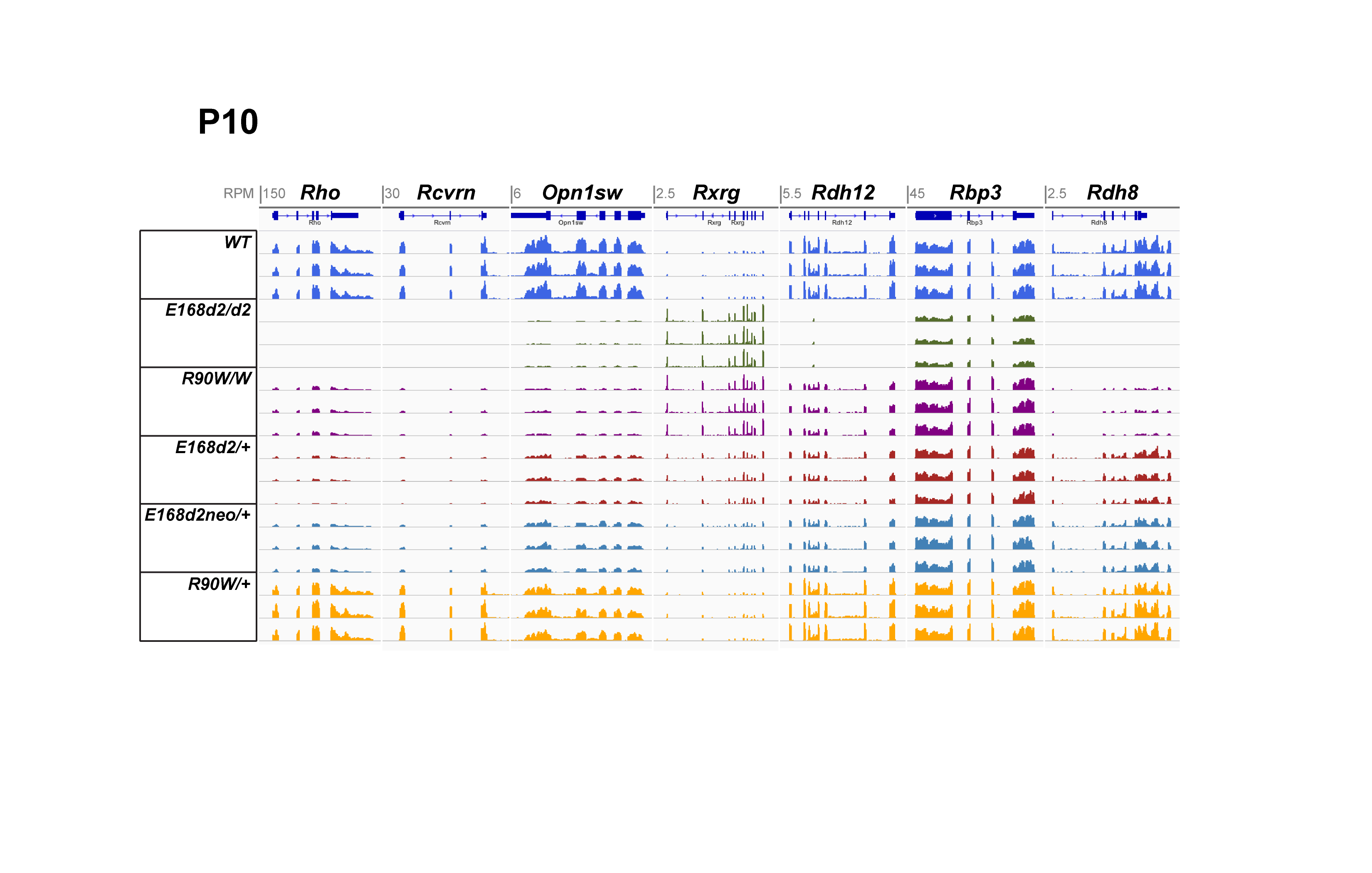

Supplement: Additional file 4: Figure S2. — P10 (a) and P21 (b) browser shots of reads per million (RPM) normalized mapped read count for selected genes for each biological replicate show reproducibility of sequencing results between samples. (ZIP 312 kb) [file 13059_2015_732_MOESM4_ESM.zip › Fig_S2A.tif]

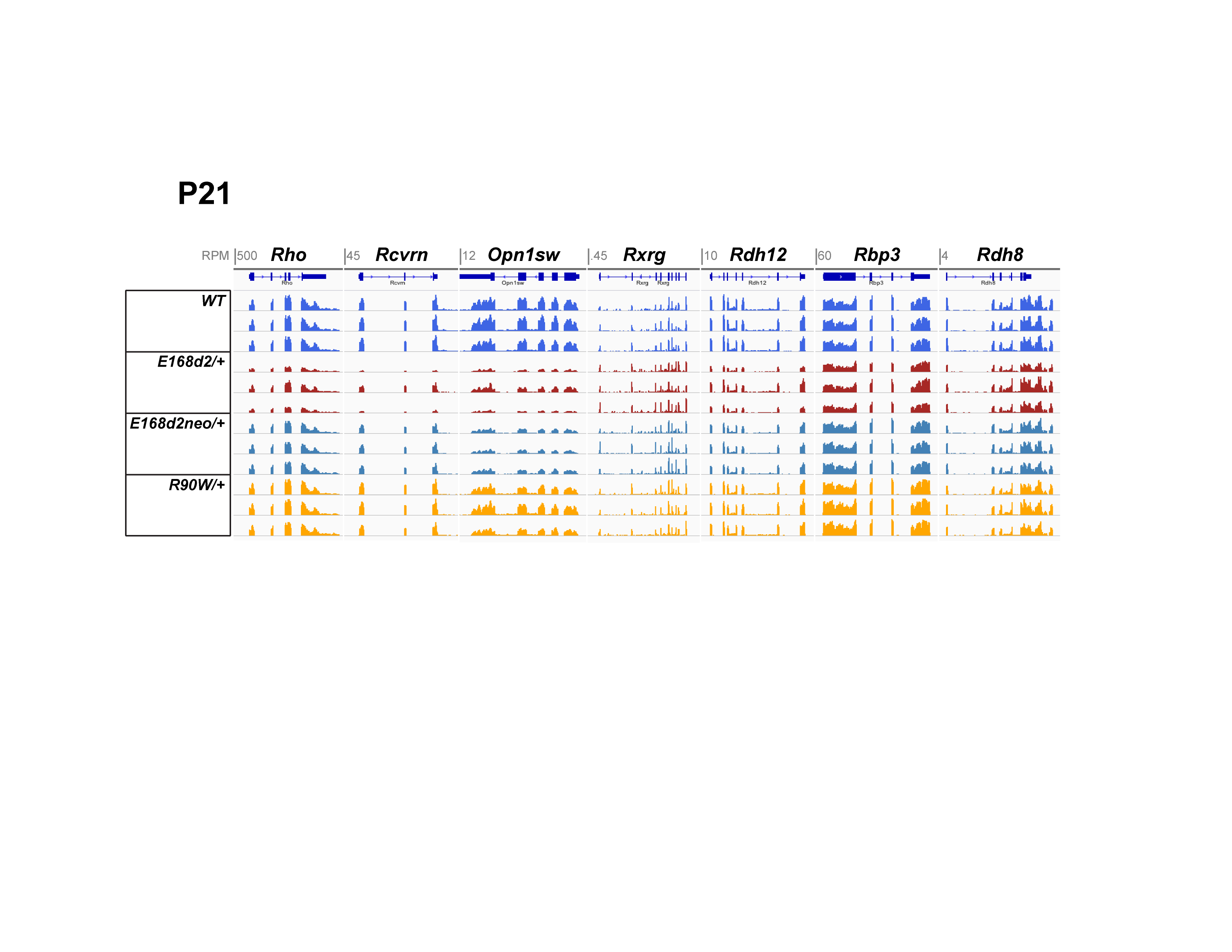

Supplement: Additional file 4: Figure S2. — P10 (a) and P21 (b) browser shots of reads per million (RPM) normalized mapped read count for selected genes for each biological replicate show reproducibility of sequencing results between samples. (ZIP 312 kb) [file 13059_2015_732_MOESM4_ESM.zip › Fig_S2B.tif]

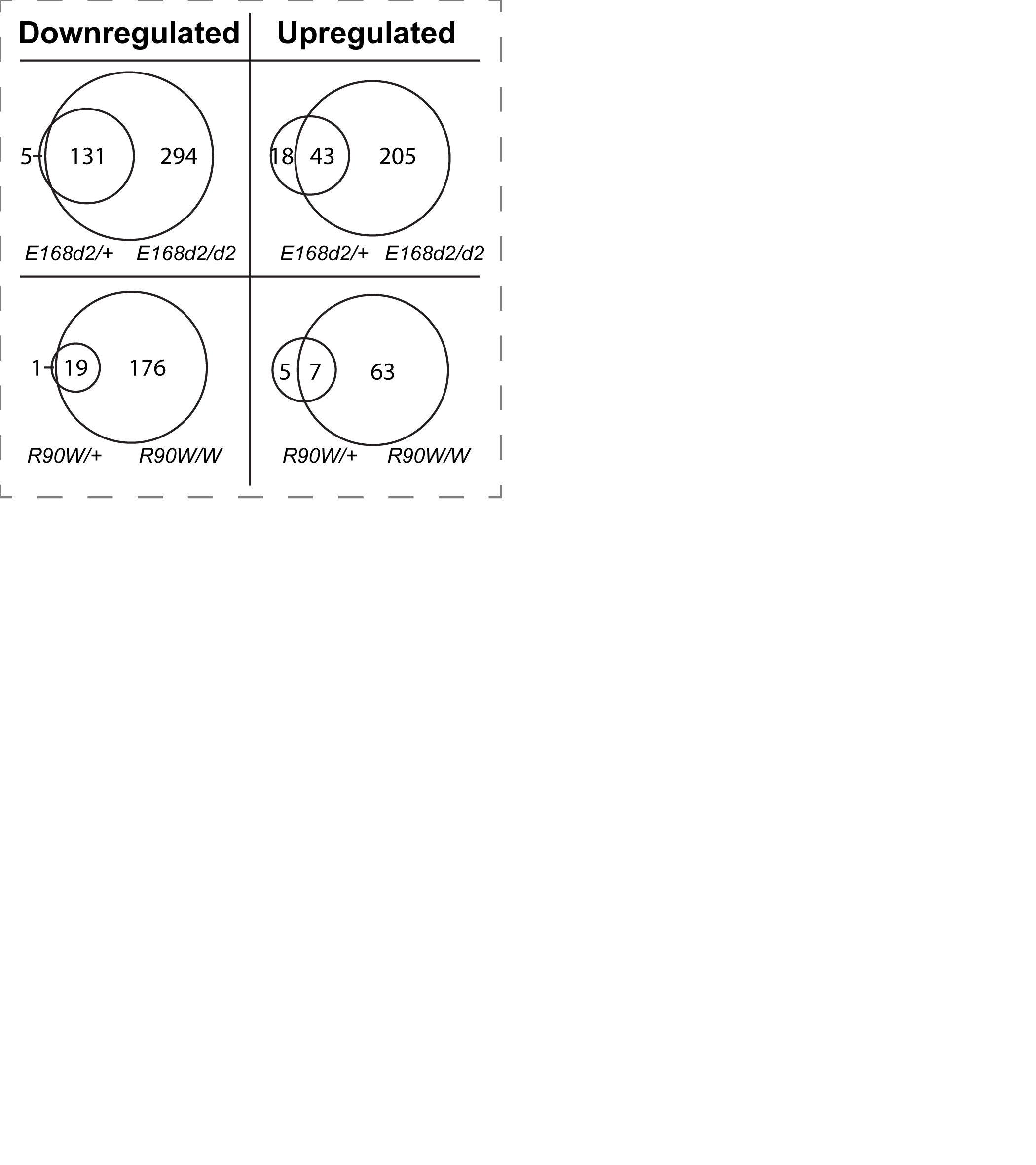

Supplement: Additional file 5: Figure S3. — Homozygous and heterozygous mutants show largely overlapping datasets. Venn diagrams comparing significantly affected transcripts (FC ≥ 2 or ≤ -2, FDR ≤ 0.05 relative to WT) in the indicated heterozygous and homozygous Crx mutant retinas. (TIFF 14588 kb) [file 13059_2015_732_MOESM5_ESM.tif]

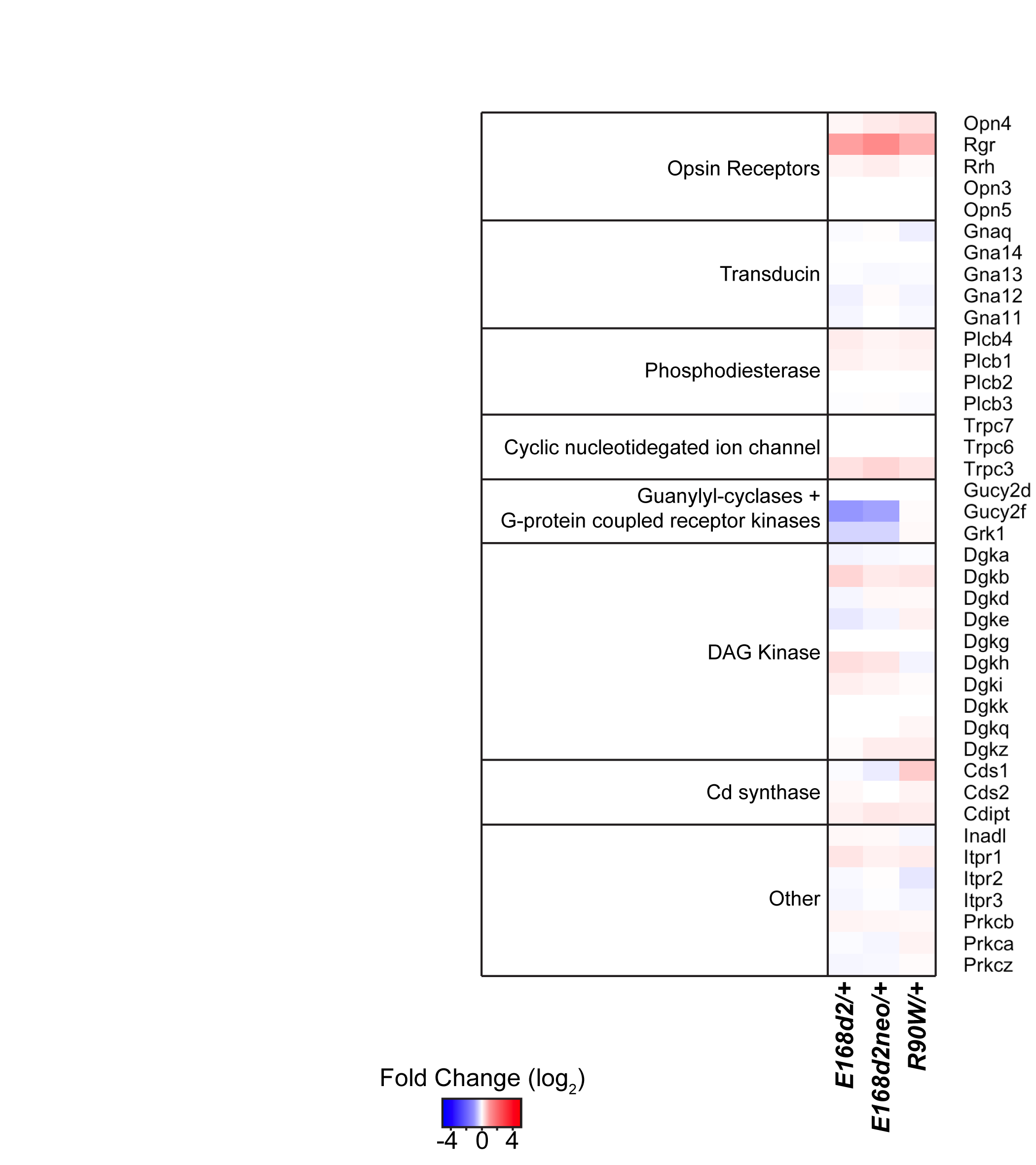

Supplement: Additional file 6: Figure S4. — Crx mutants show little change to other non-photoreceptor phototransduction components. Heatmap depicts P10 FC relative to WT for genes involved in phototransduction enriched in non-photoreceptor retinal cell types in the heterozygous mutants. (TIFF 14588 kb) [file 13059_2015_732_MOESM6_ESM.tif]

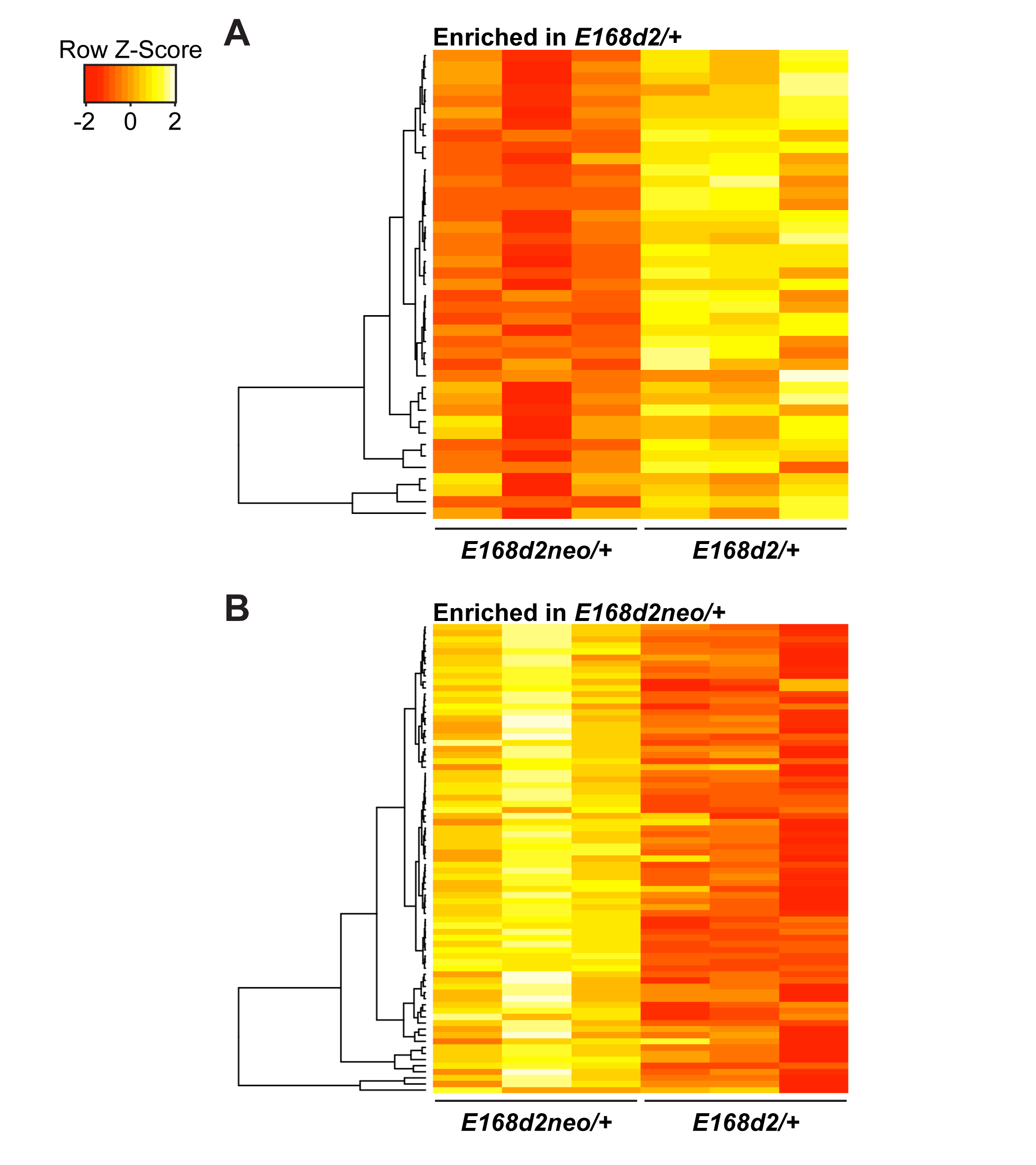

Supplement: Additional file 7: Figure S5. — Heatmap showing the Z-scores comparing three biological replicates of genes determined to be enriched in either E168d2/+ (a) or E168d2neo/+ (b) retinas. Genes are arranged in rows, clustering dendrogram is shown on the left. Patterns of expression across biological replicates are consistent, supporting the statistical significance of the difference calculated by EdgeR RNA-seq analysis. (TIFF 14588 kb) [file 13059_2015_732_MOESM7_ESM.tif]

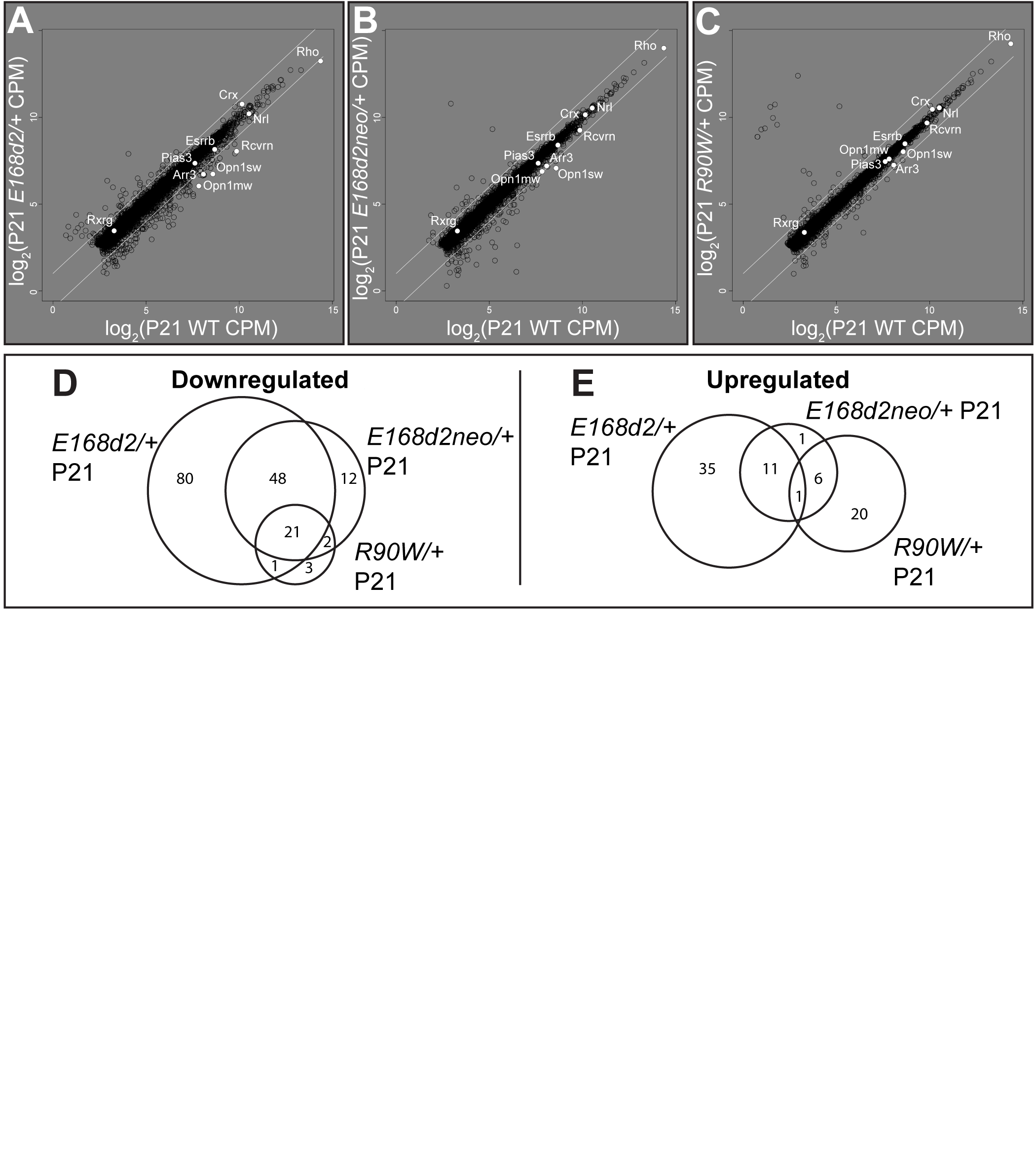

Supplement: Additional file 8: Figure S6. — Crx mutants show graded changes in gene expression of overlapping gene sets at P21. a–c Log2 CPM from P21 heterozygous E168d2/+ (a), E168d2neo/+ (b), and R90W/+ (c) mice (y-axes) are compared with log2 CPM from age-matched WT C57Bl/6 J (x-axes) mice. White letters highlight prototypical photoreceptor transcripts. White diagonal lines represent ±2 FC. d, e Venn diagrams illustrate the numbers of overlapping and distinct significantly affected genes (FC ≥ 2 or ≤ -2, FDR ≤ 0.05). (TIFF 14588 kb) [file 13059_2015_732_MOESM8_ESM.tif]

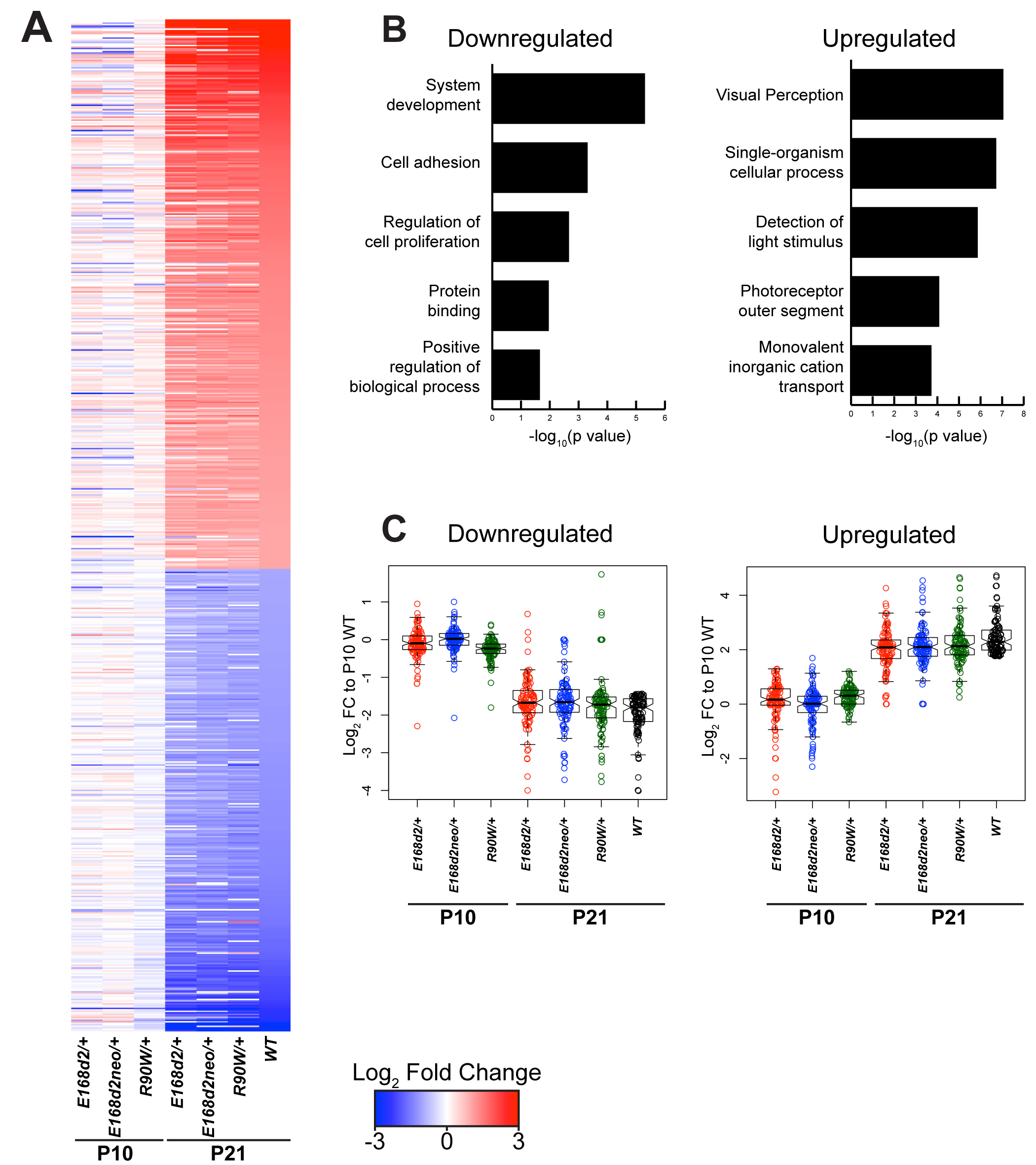

Supplement: Additional file 9: Figure S7. — Crx mutants do not abandon the developmental program, but many genes fail to reach proper expression levels. a Heatmap, ordered by the magnitude of expression change between P10 and P21 in WT retina, depicts FC compared with P10 WT to analyze developmental dynamics. b Top GO terms from analysis of top 100 genes that are down- (left panel) and up- (right panel) regulated normally during late postnatal retinal development (P21 WT versus P10 WT; FC ≤ −2 or ≥ 2, FDR ≤ 0.05). c Violin and boxplots quantifying median FC from P10 WT of each mutant. (TIFF 14588 kb) [file 13059_2015_732_MOESM9_ESM.tif]

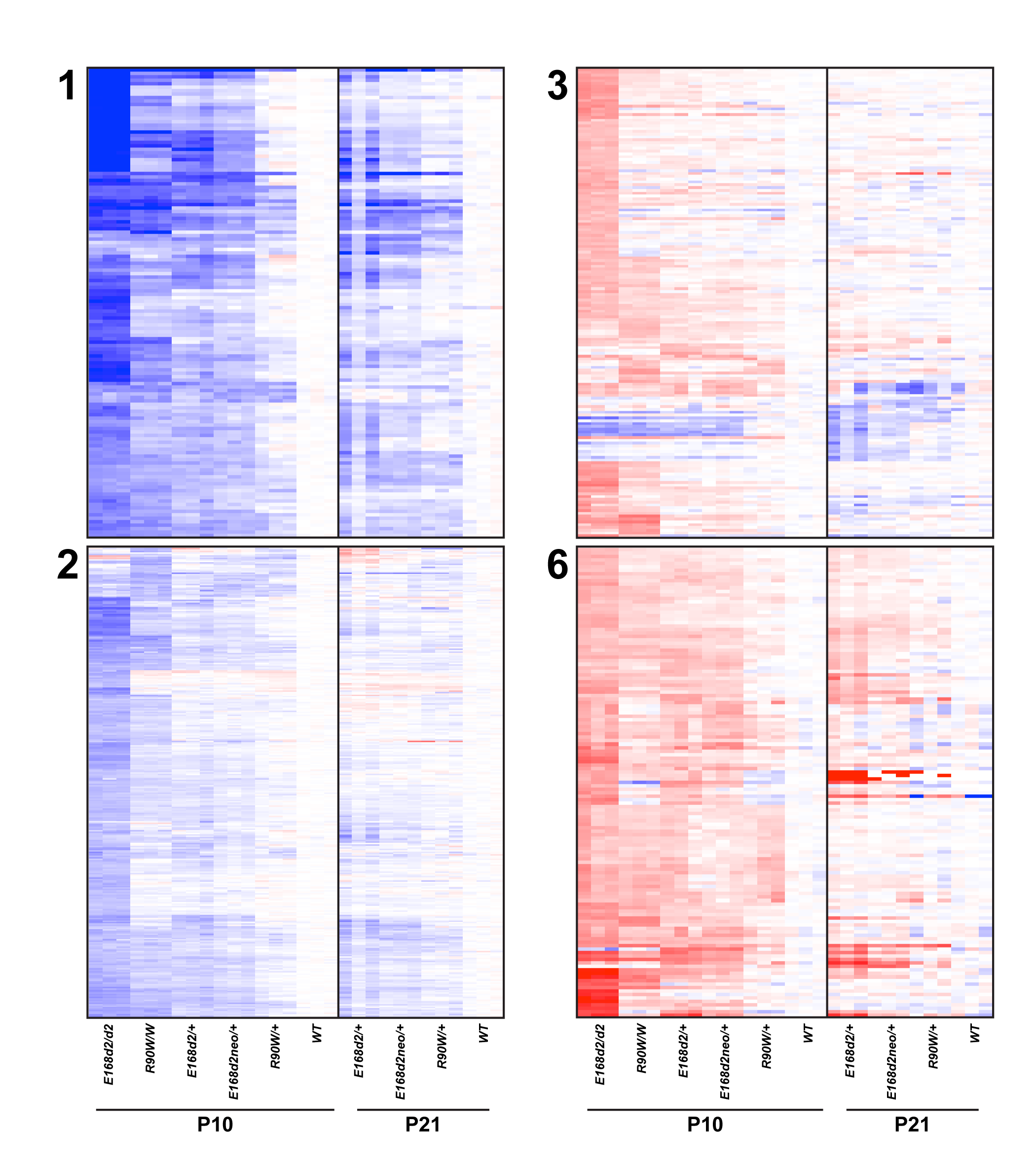

Supplement: Additional file 12: Figure S8. — Groups 1, 2, 3, and 6 genes (defined in Fig. 5) presented as a heatmap with calculated FC values for each biological replicate compared with the mean age-appropriate WT value. All genes are ordered exactly as presented in Fig. 5. (TIFF 14588 kb) [file 13059_2015_732_MOESM12_ESM.tif]

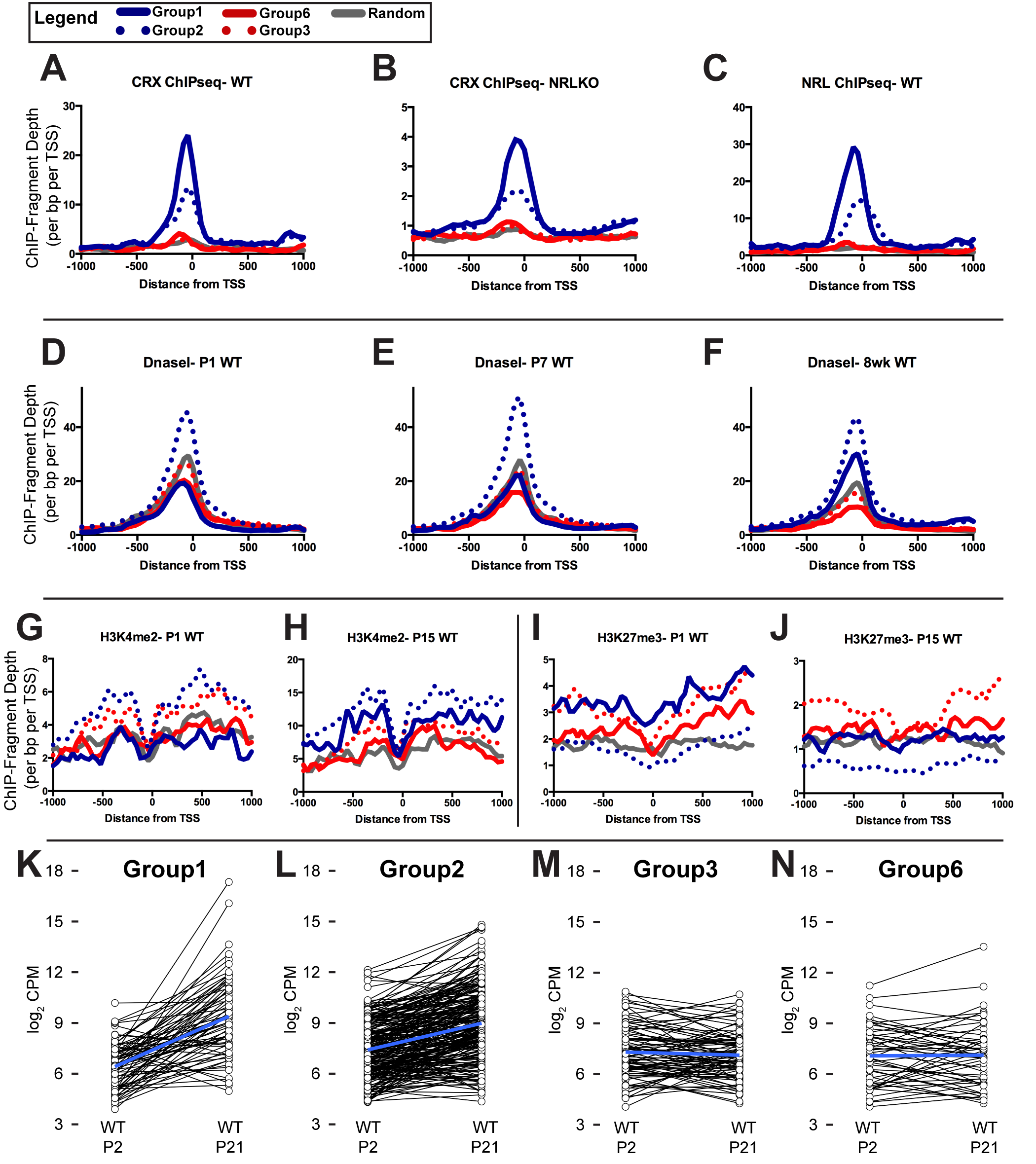

Supplement: Additional file 13: Figure S9. — Quantification of normal epigenetic marks near genes in groups 1, 2, 3, and 6 distinguishes up- and down-regulated genes in Crx mutants. Control set of equal sized random group of mm9 genes used as background control (grey in all). a–c Quantification of CRX ChIP-seq [15] in rod-dominant WT retina (a) and cone-dominant Nrl−/− retina (b), and NRL ChIP-seq [17] in WT retina (c). d–f DNase I hypersensitivity plots from WT retina at the three indicated ages [22]. g, h H3K4me2 ChIP-seq of WT retina at the two ages [23]. i, j H3K27me3 ChIP-seq of WT retina at the two ages. All represent normalized mean read depth of each library centered on the TSS of genes within groups 1, 2, 3, and 6. k–n Analysis of WT expression of each gene and average change in expression (blue line) from the age of P2 to P21 (raw data in [24]), presented as log2 CPM. (TIFF 14576 kb) [file 13059_2015_732_MOESM13_ESM.tif]

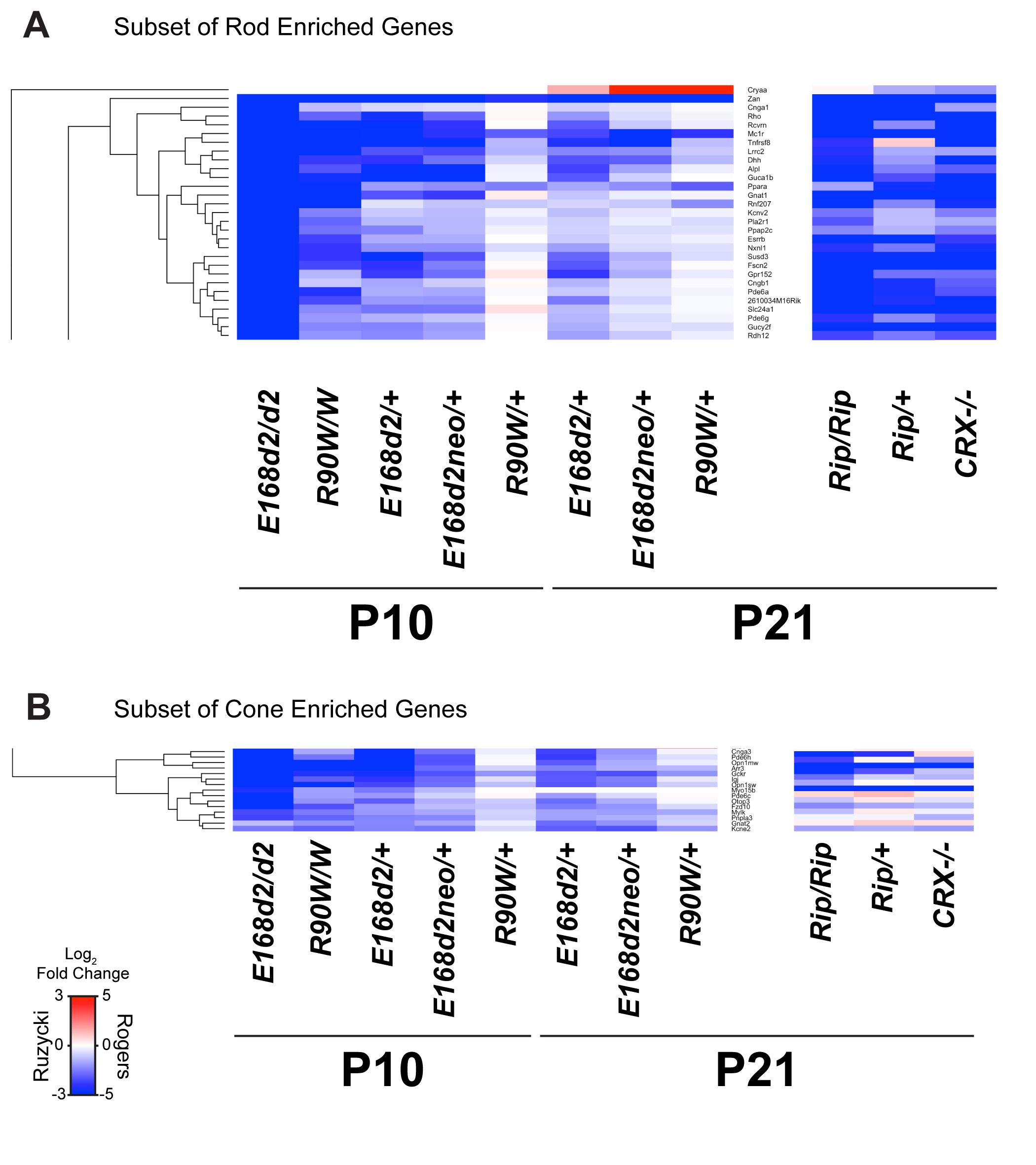

Supplement: Additional file 14: Figure S10. — Expanded view of areas marked with asterisks in Fig. 6. All data and presentation exactly as described previously. (TIFF 14588 kb) [file 13059_2015_732_MOESM14_ESM.tif]

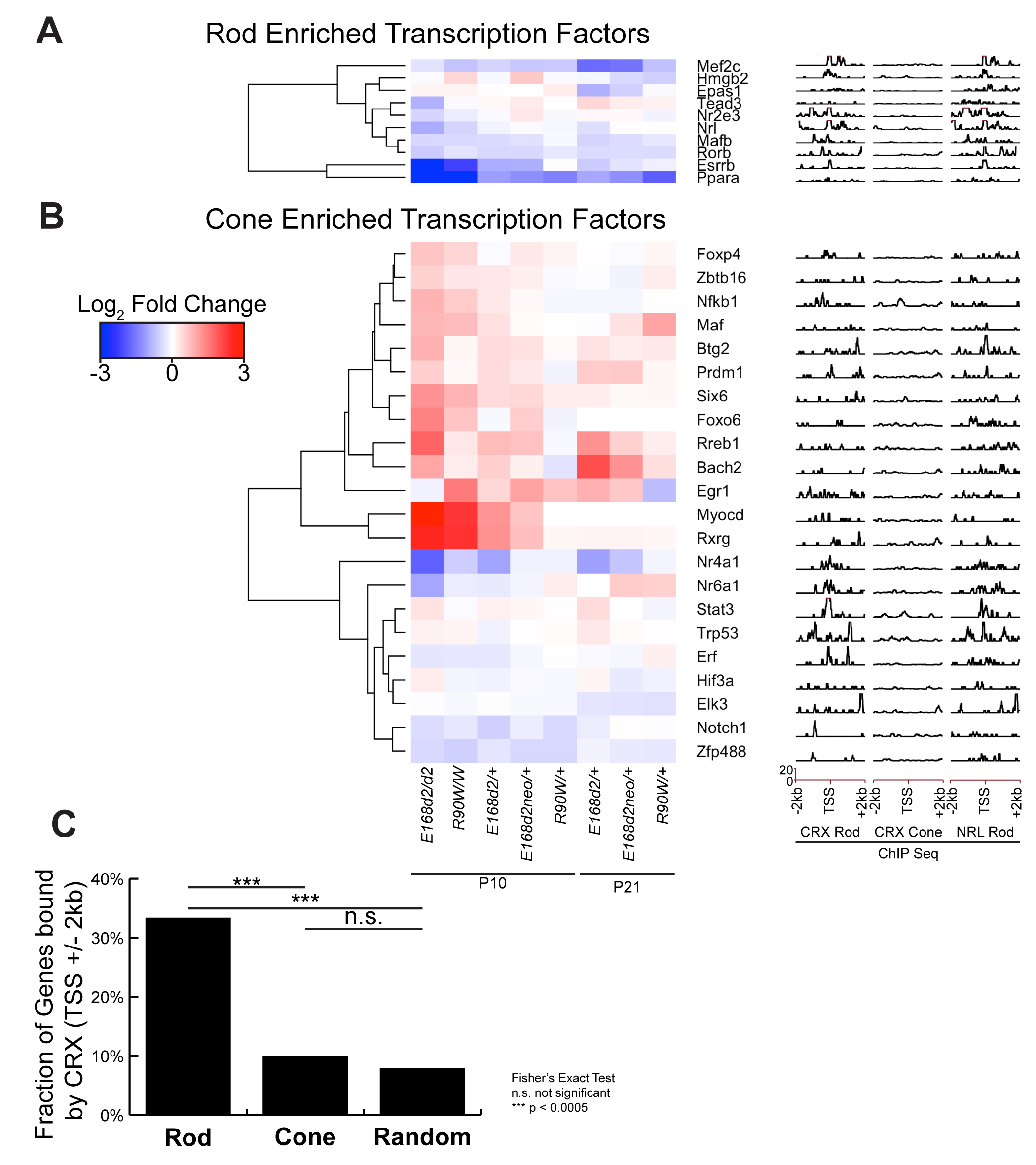

Supplement: Additional file 15: Figure S11. — Changes in rod- and cone-enriched transcription factors are consistent with general patterns of rod and cone gene expression in Crx mutants. a, b Heatmap describes FC relative to WT in each Crx mutant for rod- (a) and cone-enriched (b) genes annotated as transcription factors (raw data in [24]; WT versus Nrl−/− RNA-seq FC ≤ −2 or ≥2 and FDR ≤ 0.05; GO:0003700). ChIP-seq data are presented for 4-kb windows surrounding the TSS for each gene for CRX (in rods and cones) and NRL (in rods). c Analysis detailing percent of rod-enriched, cone-enriched, or random set of genes with CRX ChIP-seq peak within 2 kb of TSS in WT retina. Note the significant enrichment of CRX binding in the rod set over cone and random gene sets. (TIFF 14588 kb) [file 13059_2015_732_MOESM15_ESM.tif]

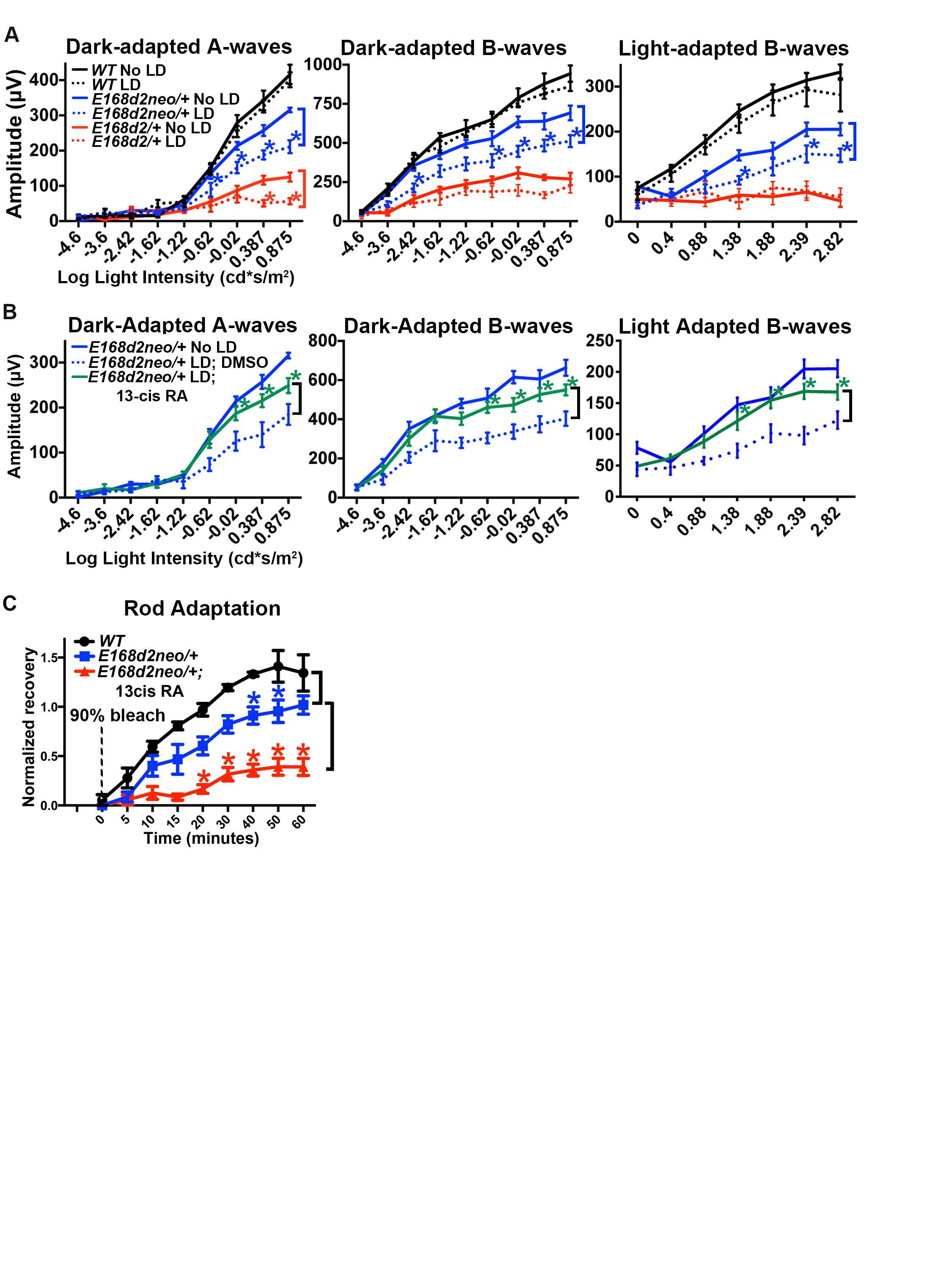

Supplement: Additional file 16: Figure S12. — Retinal function is affected by light damage (LD) in E168d2neo/+ but not E168d2/+ mutant mice. a Intensity-response plots for dark-adapted ERG a-wave, dark-adapted b-wave and light-adapted b-wave from mice with the indicated genotypes, with or without LD treatment. b Intensity-response plots for ERG responses following LD for mice pretreated with either 13-cis-RA or DMSO. c Rod dark adaptation measured by recovery of maximal rod ERG a-wave following >90 % rhodopsin photobleach for WT and E168d2neo/+ mice, with and without drug treatment. Error bars represent standard error of mean (n ≥ 3). Asterisks mark data points of significant difference (p ≤0.05) from the control as determined by two-way ANOVA (see "Materials and methods"). (TIFF 3018 kb) [file 13059_2015_732_MOESM16_ESM.tif]

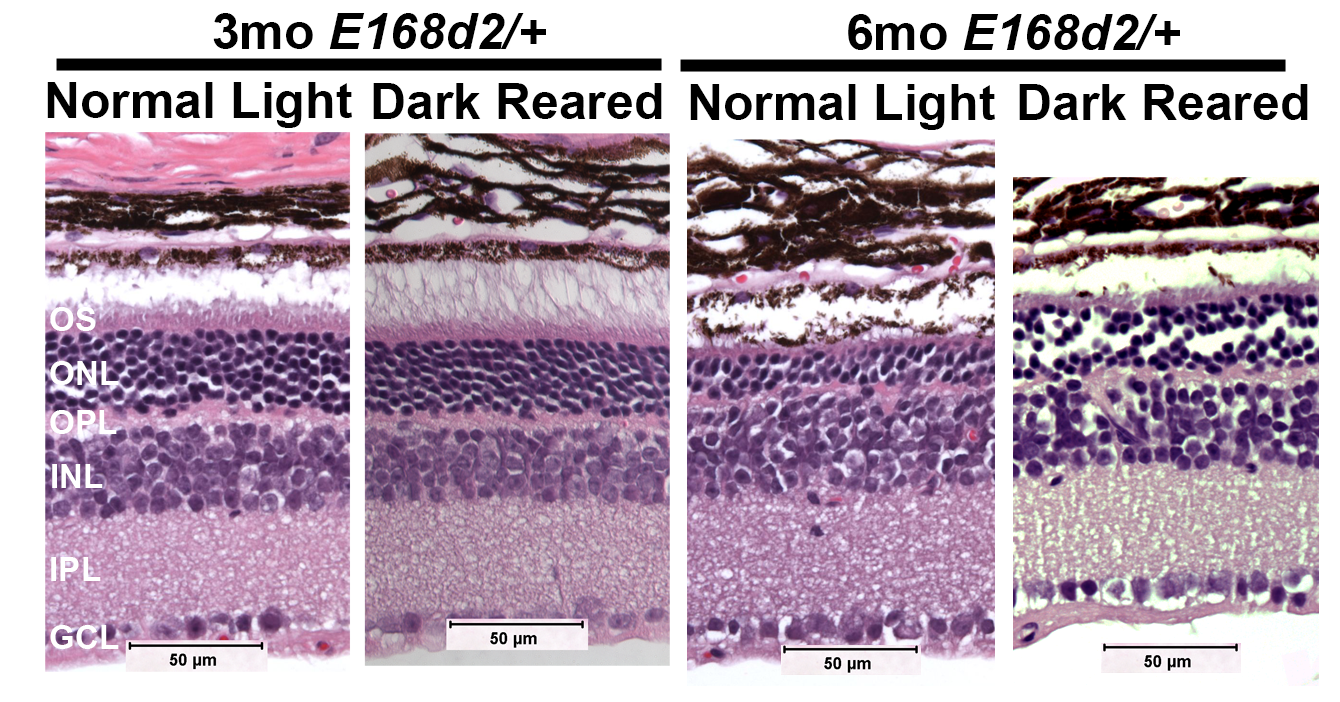

Supplement: Additional file 17: Figure S13. — E168d2/+ photoreceptor degeneration is light-independent. Retinal morphology of E168d2/+ mice raised under 12 h light–dark cycle (normal light) or constant darkness (dark-reared) for 3 or 6 months. Note that the dark rearing did not improve ONL thinning in the mutant retina. GCL ganglion cell layer, INL inner nuclear layer, IPL inner plexiform layer, OS outer segment, ONL outer nuclear layer, OPL outer plexiform layer. Scale bar = 50 μM. (TIFF 3603 kb) [file 13059_2015_732_MOESM17_ESM.tif]

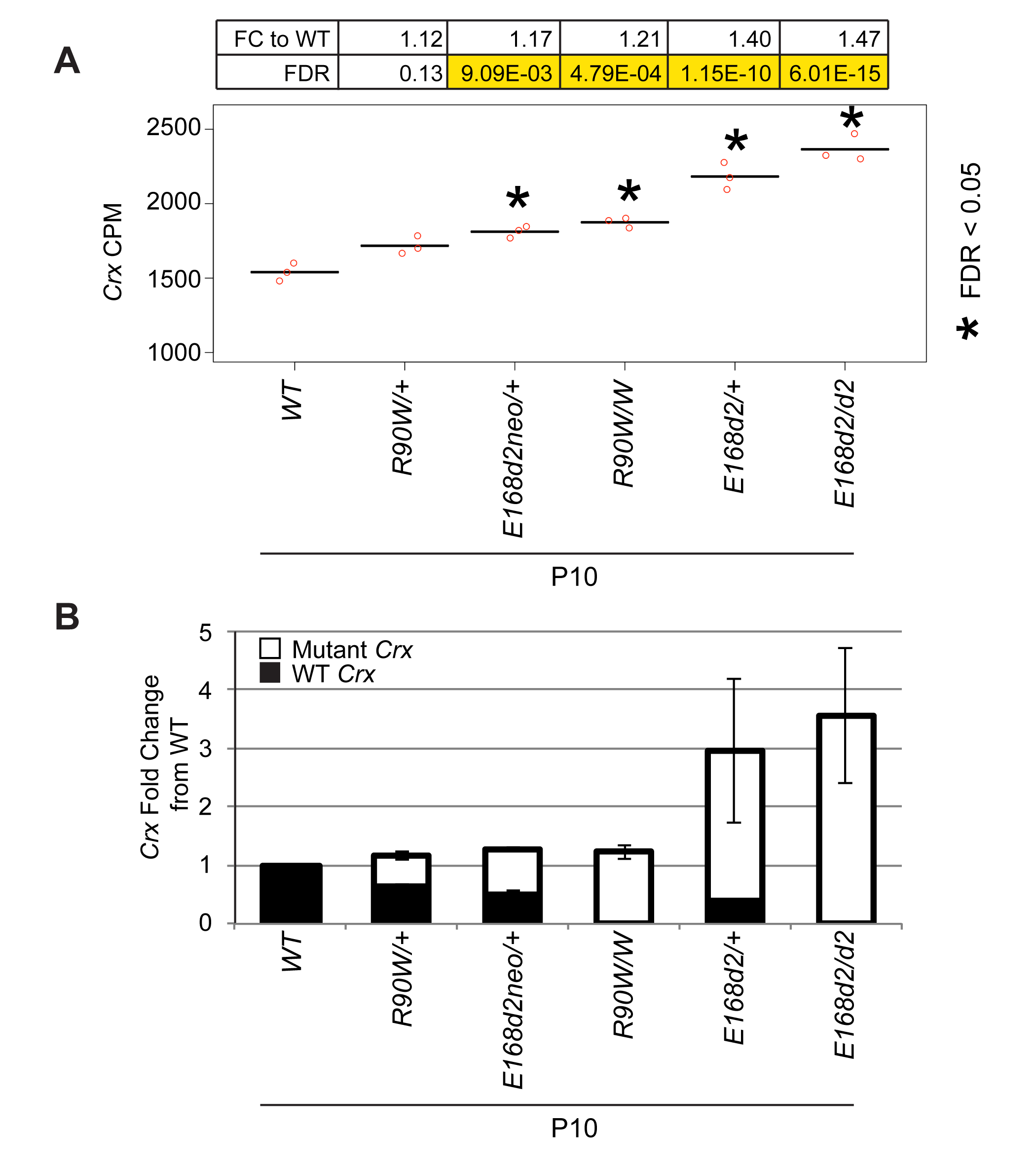

Supplement: Additional file 18: Figure S14. — Increased Crx expression in mutant lines at P10. a RNA-seq derived raw CPM values for each biological replicate (red circles) and mean (black bar). Top panel of spreadsheet lists the EdgeR calculated FC and FDR for each genotype relative to WT control. Asterisks denote samples where FDR < 0.05. b qRT-PCR analysis of transcript levels of WT and mutant Crx alleles in each genotype. Note the same trend of Crx expression changes in these mutants detected by both methods. (TIFF 14588 kb) [file 13059_2015_732_MOESM18_ESM.tif]
